# The role of long noncoding RNAs in ocular angiogenesis and vascular oculopathy

**DOI:** 10.1186/s13578-024-01217-5

**Published:** 2024-03-23

**Authors:** Pranali Gandhi, Yuzhi Wang, Guigang Li, Shusheng Wang

**Affiliations:** 1 Tulane University School of Medicine, New Orleans, LA 70112 USA; 2 Louisiana State University School of Medicine, New Orleans, LA 70112 USA; 3 Department of Ophthalmology, Tongji Hospital, Tongji Medical College, Huazhong University of Science and Technology, Wuhan, Hubei province P.R. China; 4 Department of Cell and Molecular Biology, Tulane University, New Orleans, LA 70118 USA; 5 Department of Ophthalmology, Tulane University, New Orleans, LA 70112 USA; 6 Tulane Personalized Health Institute, Tulane University, New Orleans, LA 70112 USA

**Keywords:** Ocular, Eye disease, Angiogenesis, Long noncoding RNA (lncRNA)

## Abstract

**Background:**

Long noncoding RNAs (lncRNAs) are RNA transcripts over 200 nucleotides in length that do not code for proteins. Initially considered a genomic mystery, an increasing number of lncRNAs have been shown to have vital roles in physiological and pathological conditions by regulating gene expression through diverse mechanisms depending on their subcellular localization. Dysregulated angiogenesis is responsible for various vascular oculopathies, including diabetic retinopathy, retinopathy of prematurity, age-related macular degeneration, and corneal neovascularization. While anti-VEGF treatment is available, it is not curative, and long-term outcomes are suboptimal, and some patients are unresponsive.

**Results and summary:**

To better understand these diseases, researchers have investigated the role of lncRNAs in regulating angiogenesis and models of vascular oculopathies. This review summarizes recent research on lncRNAs in ocular angiogenesis, including the pro-angiogenic lncRNAs *ANRIL*, *HOTAIR*, *HOTTIP*, *H19*, *IPW*, *MALAT1*, *MIAT*, *NEAT1*, and *TUG1*, the anti-angiogenic lncRNAs *MEG3* and *PKNY*, and the human/primate specific lncRNAs *lncEGFL7OS*, discussing their functions and mechanisms of action in vascular oculopathies.

## Introduction

Angiogenesis is a fundamental process during embryogenesis and under physiological and pathological conditions in adults. It is an endogenous mechanism to create new blood vessels from a pre-existing vascular network, which is distinct from vasculogenesis by which the blood vessels are formed de novo [1]. In adults, angiogenesis is tightly regulated by hypoxia, flow stress, and an array of pro- or anti-angiogenic factors to control normal physiological growth, reproduction, wound repair, and response to ischemia. Dysregulation of angiogenesis could result in pathological conditions, including tumor growth and metastasis, and vascular oculopathies. Angiogenesis involves the activities of multiple cell types, including endothelial cells (EC), vascular smooth muscle cells (VSMC), and pericytes. Small blood vessels like capillaries are lined internally by a single layer of ECs and wrapped by pericytes embedded in the base membrane, while the larger blood vessel EC layer is covered by a VSMC layer and connective tissue. The EC layer of the blood vessels acts as a mediator of molecule exchange, cytokine secretion, cell extravasation, permeability, and vascular tone control at the blood-tissue interface [2]. During angiogenesis, the capillary basement membrane is degraded, and the ECs migrate and proliferate to form primary capillaries. The newly formed blood vessels serve to meet the increasing oxygen demand of growing tissues. Two types of angiogenesis exist in the body, including sprouting angiogenesis and intussusception. Sprouting angiogenesis is driven by the migration of the “tip cells” and the proliferation of the “stalk cells”, which guide capillaries to sprout and form lumens in response to a gradient of angiogenic factors. Intussusception is the splitting of pre-existing vessels into new ones.

Numerous angiogenic factors have been shown to regulate angiogenesis. One of the most studied angiogenic factors is vascular endothelial growth factor (VEGF)-A, an essential growth factor specifically for ECs. VEGF-A is activated by hypoxia-inducible transcription factors (HIFs), which become stable under low oxygen conditions. VEGF-A binds to its receptors VEGFR1 (Flt1) and VEGFR2 (Flk1 or KDR) on the cell membrane of ECs, and activates downstream signaling pathways, including PI3K/AKT, Ras/MAPK, FAK/Paxillin, and PLCγ pathways, to regulate cell survival, proliferation, cytoskeletal rearrangement, and vascular permeability, respectively. This drives angiogenesis and enhances vascular permeability. Other growth factors involved in angiogenesis include but are not limited to platelet-derived growth factor (PDGF), placental-derived growth factor (PlGF), fibroblast-growth factor (FGF), and angiopoietin (Ang)-1 and − 2. PDGF signals through PDGF receptors PDGFRα and PDGFRβ and induces angiogenesis by up-regulating VEGF and promoting the proliferation and recruitment of perivascular cells [3]. PlGF stimulates angiogenesis by binding to coreceptors Neuropilin 1 and 2 (NRP1 and NRP2) and VEGFR1 directly or by interacting with VEGF [4]. FGF is a broad-spectrum mitogen that promotes numerous activities including angiogenesis and tumorigenesis [5]. Ang-1 is a potent angiogenic growth factor signaling through its receptor Tie-2 on the EC surface [6]. It is critical for vessel maturation and mediates EC migration, adhesion, and survival. Ang-2 was initially found to disrupt the connections between EC and perivascular cells and promote vascular regression. However, later studies indicate context-dependent functions of Ang-2. In conjunction with VEGF, Ang-2 promotes neovascularization. Therefore, multiple growth factors and signaling pathways are involved in the regulation of angiogenesis.

## Ocular angiogenesis, vascular oculopathies and current treatments (table 1)

**Table 1:** Vascular Oculopathies and Current Treatments

Vascular Oculopathies	Disease Features	Current Treatments	Refs
Corneal Neovascularization (KNV)	Peri-corneal vessels grow and invade the stroma obstructing vision, causing blindness.	Laser photocoagulation, topical steroid treatments	[8]
Diabetic Retinopathy (DR)	Retinal neovascularization in proliferative DR, which could progress to vitreous hemorrhage or tractional retinal detachment, leading to blindness.	Laser photocoagulation, Anti-VEGF therapy	[10]
Age-related Macular Degeneration (AMD)	Exudation, choroidal neovascularization (CNV) and retinal neovascularization in the macula in wet AMD, leading to hemorrhage and acute blindess.	Anti-VEGF or anti-VEGF/Ang2 therapy, Laser photocoagulation (less common)	[13, 14]
Sickle Cell Retinopathy (SCR)	Chronic vaso-occlusion. Non-proliferative (NP) SCR usually does not cause vision loss whereas proliferative PSCR leads to neovascularization, infarcts, vitreous hemorrhages, or retinal detachments in severe cases.	Laser photocoagulation, Off-label anti-VEGF, Hydroxyurea, red blood cell exchange transfusion	[11, 138]
Retinopathy of Prematurity (ROP)	Retinal neovascularization in prematurely born babies, causing mild vision loss.	Laser photocoagulation, Anti-VEGF therapy	[9]

The eye has a unique vascular structure, with different segments having varying levels of vascularity. The cornea is known as a “privileged” tissue because it is normally avascular, which is crucial for maintaining optical clarity and protecting it from immune recognition [7]. However, insults such as hypoxia, infection, inflammation, corneal transplant rejection, or trauma, can disrupt the balance of pro- and anti-angiogenic factors around and in the cornea and lead to pathological corneal vascularization. **Corneal neovascularization (KNV)** occurs when peri-corneal vessels grow and invade the stroma obstructing vision, which is a significant cause of blindness [8].

The vasculature of the retina also plays an essential role in vision, supplying oxygen and nutrients for the inner retina. The choroid vasculature, a dense vascular network between the retinal pigment epithelium (RPE) and the sclera, supplies the RPE and the outer retina (mainly the photoreceptors) [9]. The macula, an oval-shaped yellowish area surrounding the fovea at center of the retina, is required for our central vision and most of our color vision. Notably, the macula normally doesn’t have blood vessels on its inner surface. The inner retinal vasculature is affected by diabetes, hypertension, premature birth, and sickle cell disease.

**Diabetic retinopathy (DR)** is a neurovascular disease of the retina that is caused by hyperglycemia, leading to damage in the retinal blood vessels causing them to leak fluid, exudates, microaneurysms, and hemorrhages, defined as non-proliferative DR (NPDR). Progression of damage causes a hypoxic state in the retina which induces neovascularization, termed proliferative DR (PDR) [10]. If PDR advances, it could progress to vitreous hemorrhage or tractional retinal detachment, leading to blindness.

In **sickle cell disease (SCD)**, a recessive point mutation in the hemoglobin gene locus creates crescent shaped red blood cells, causing chronic vaso-occlusive events that can affect the eye in various ways. SCD can cause retinal artery occlusion, ischemic optic neuropathy, and SCD maculopathy, and most commonly, non-proliferative and proliferative sickle cell retinopathy (NPSCR and PSCR) [11]. NPSCR usually does not cause vision loss whereas PSCR leads to neovascularization, infarcts, vitreous hemorrhages, or retinal detachments in severe cases.

Retinal neovascularization can also occur in prematurely born infants. Infants born before 31 weeks of gestation or weigh under 1250 g are at high risk for developing **retinopathy of prematurity (ROP).** ROP is a unique vascular disease which has a combination of inadequate physiological blood vessel development and pathological blood vessel formation. During ROP, vessels sprout from the retina into the vitreous causing mild vision loss in 90% of newborns [9, 12].

**Age-related macular degeneration (AMD)** is a leading cause of elderly population blindness. Late AMD has two forms, “dry” and “wet” AMD. Dry AMD is distinguished by drusen deposits between the RPE and Bruch’s membrane. Wet AMD could occur dependently or independently of dry AMD and is characterized by exudation, choroidal neovascularization (CNV) and retinal neovascularization in the macula [13, 14]. Progressive damage to Bruch’s membrane induces neovascularization via upregulation of VEGF in the choroid. This leads to the outgrowth of abnormal choroidal vessels underneath the RPE, resulting in subretinal extravasations leading to hemorrhage before they regress and form a scar.

As neovascularization in the cornea, retina and choroid could lead to blindness, anti-angiogenic and anti-inflammatory approaches, including anti-VEGF, laser/phototherapy, corticosteroids and nonsteroidal anti-inflammatory drugs (NSAIDs), are currently used to treat these conditions. Anti-VEGF agents, including Macugen, Lucentis and Eylea, are the current mainstay for treating wet AMD [15–17]. They have shown markedly improved clinical outcomes in most wet AMD patients [18, 19]. However, it is not a cure for AMD. After seven years of treatment, only a third of the patients demonstrated good vision outcomes [20]. Recently a bi-specific antibody to VEGF and Ang2, faricimab, has been approved by the FDA to treat wet AMD and diabetic macular edema (DME) [21, 22]. Due to its prolonged activity, faricimab allows for injection every three or four months in wet AMD and DME patients instead of the monthly injection for other anti-VEGF antibodies. Anti-VEGF drugs have also proven to be beneficial for patients with DR with or without diabetic macular edema, KNV and ROP. However, some patients are only partially responsive to these agents [23–25].

Besides anti-VEGF drugs, other treatment options are also used for ocular neovascularization. While laser photocoagulation is effective for KNV and is still the golden standard for DR, it is not commonly used for wet AMD treatment due to potential for retinal damage in laser photocoagulation and the higher efficacy of anti-VEGF drugs [10, 26]. Topical steroid treatments have also been effective for KNV. Steroid implants and NSAIDs are in clinical trials for ocular neovascular diseases. Although current therapies work well for preventing further disease progression and maintaining vision, there is room for improvement. For example, anti-VEGF doesn’t treat the underlying causes of the ocular diseases and therefore failed to prevent dry AMD development. Anti-VEGF needs frequent intravitreal injections to be effective. Steroids could have side effects including glaucoma. Therefore, current research is focusing on novel therapies or delivery methods, including gene therapy, specific MMP inhibitors, oligonucleotides, nanoparticles, and stem cell therapies, are being developed [7, 8]. A deeper understanding of these diseases is the key to identifying better therapeutic targets.

## LncRNAs and their functional mechanisms

It is now accepted that the majority of mammalian genomes are transcribed but many transcripts do not encode proteins. Long non-coding RNAs (lncRNAs) represent RNA transcripts that are longer than 200 nucleotides that are not translated to proteins. However, some lncRNAs do encode micropeptides (e.g., myoregulin) [27]. The current estimated number of human lncRNAs is about 30,000 to 60,000, which is more than the number of protein coding genes [28]. Some lncRNAs are not linear but form covalently closed circle (circRNAs) and have shown functions in ocular angiogenesis. These lncRNAs are summarized well recently and will not be discussed here [29]. LncRNAs are involved in a wide range of cellular processes from development to disease, yet the functions of most lncRNAs are still unknown. LncRNAs are similar to mRNAs as they are transcribed by RNA polymerase II, usually 5′-capped, spliced, and polyadenylated. LncRNAs tend to be shorter than mRNAs, have fewer but longer exons, show lower expression levels, poor sequence conservation, and more specific expression patterns than mRNAs.

**Fig. 1:**
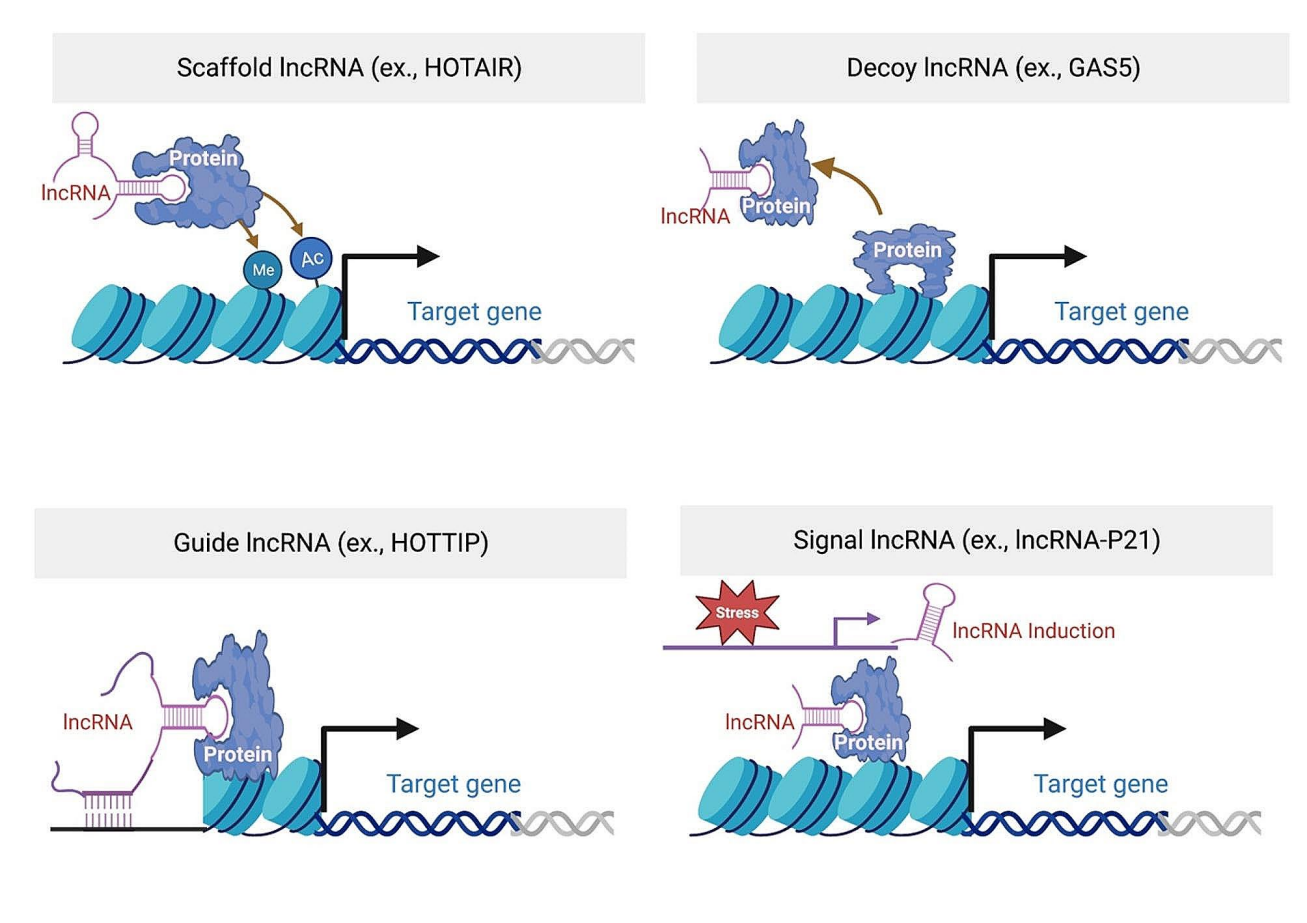
Nuclear Mechanisms of lncRNAs. Scaffold lncRNA can serve as a structural scaffold for histone or DNA modification enzymes to regulate target gene transcription. Decoy lncRNA can act as decoy by sequestering transcription factor away from its promoter region. Guide lncRNA can guide transcription factor to its promoter region by binding to both DNA and the transcription factor. Signal lncRNAs can be induced by signals or stressors and attract transcription factors to its target gene promoter region

**Fig. 2:**
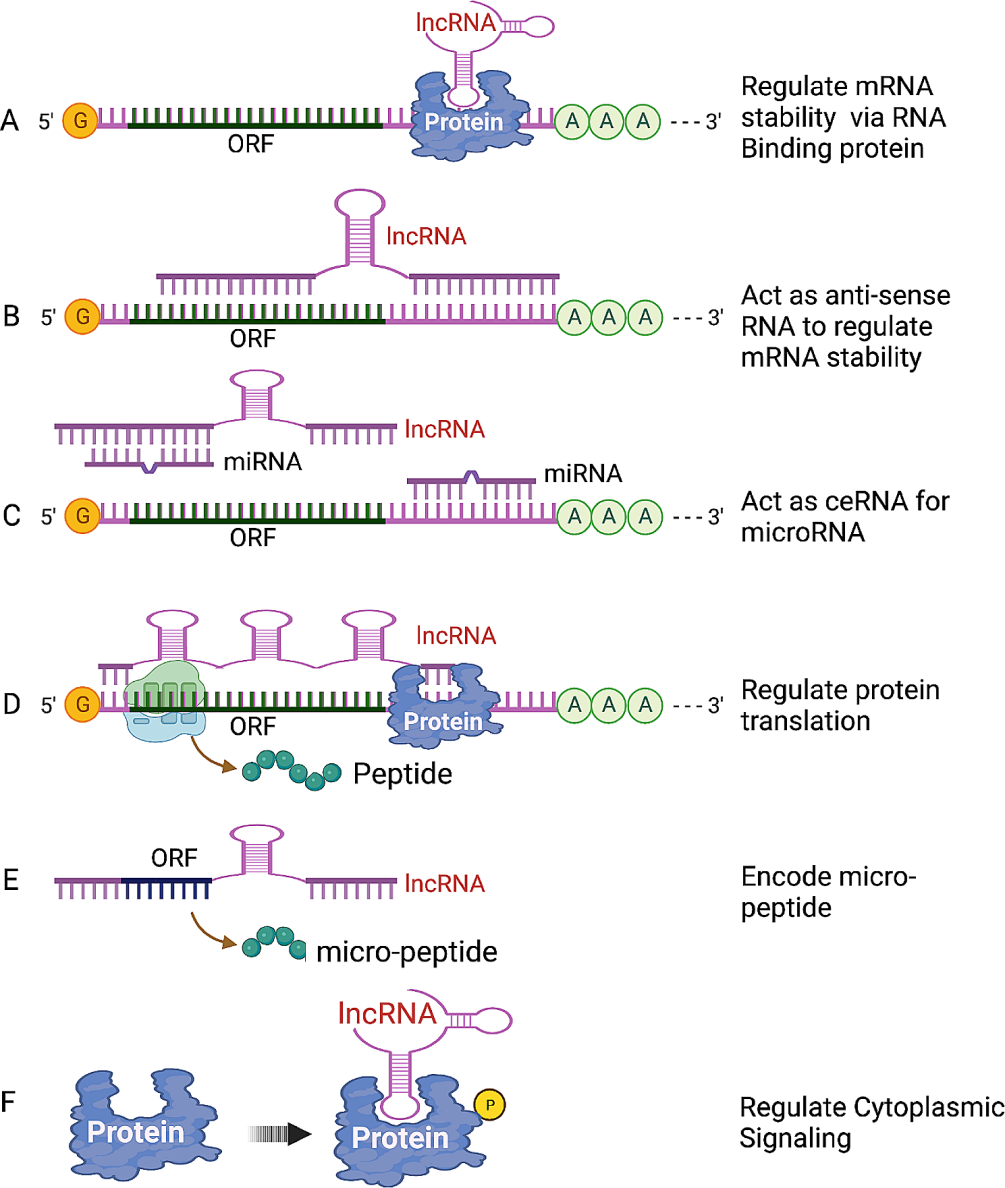
Cytoplasmic mechanisms of LncRNAs. A) LncRNAs can interact with 3’-untranslated region (UTR) binding proteins which impact mRNA stability. **B**) Antisense lncRNAs regulate mRNA stability by forming duplexes with mRNA through complementary base pairing **C**) LncRNAs can have microRNA (miRNA)-complementary sites that act as competitive endogenous RNAs (ceRNAs) and sequester miRNAs away from their targets **D**) LncRNAs regulate protein translation and affect post-translational mechanisms. **E**) LncRNAs code for regulatory micro-peptides. **F**) LncRNAs regulate signaling pathways in the cytoplasm

LncRNAs are more often localized in the nucleus than in the cytoplasm [30, 31]. In the nucleus, lncRNAs can function *in cis* or *in trans* to influence chromatin remodeling and gene expression via several mechanisms. LncRNAs working *in cis* exert their functions near to where they were transcribed in the genome, whereas trans-acting lncRNAs exert their effects in loci far away or on other regulatory RNAs and proteins [32]. Overall, nuclear lncRNAs can both inhibit and activate gene expression through different mechanisms: scaffold, decoy, guide, and signal [33] (Fig. 1). Scaffold lncRNAs carry out epigenetic modifications by acting directly on chromatin or recruiting other chromatin remodeling factors. Some examples of scaffold lncRNAs include *XIST* and *HOTAIR* which can directly or indirectly interact with DNA methyltransferase or histone methylase to regulate gene expression. Decoy lncRNAs function by sequestering the protein away from the DNA to prevent gene transcription. For example, lncRNA *Gas5* contains a hairpin motif that resembles and binds the DNA-binding domain site of the glucocorticoid receptor, therefore helping its release from DNA [33]. Guide lncRNAs act as guides to target chromatin-modifying complexes to specific genomic locations to regulate gene expression through either RNA-DNA or RNA-protein-DNA interactions. For example, *HOTTIP* directly binds to the adaptor protein WDR5, targeting it to the HOXA locus and regulating its transcription [34]. Lastly, lncRNAs can also act as signals for activation of transcription or translation of genes and other regulatory RNAs. *LncRNA-p21* is induced by p53 and interacts with nuclear heterogeneous ribonucleoprotein-K to inhibit the expression of p53 downstream genes [35]. Cytoplasmic lncRNAs can regulate mRNA stability and translation (Fig. 2). LncRNAs can interact with 3’-untranslated region (UTR) binding proteins that affect mRNA stability. Antisense lncRNAs can regulate mRNA stability by forming duplexes with mRNA. Certain lncRNAs have microRNA (miRNA)-complementary sites that act as competitive endogenous RNAs (ceRNAs) or “sponges” for miRNAs, therefore decreasing the ability of miRNAs to target mRNA. Some lncRNAs regulate protein translation and affect post-translational mechanisms. Some lncRNAs code for regulatory micro-peptides. LncRNAs can also regulate signaling pathways in the cytoplasm [33]. Of note, the functional modes of lncRNAs are not exclusive to each other, as one lncRNA could exploit multiple modes of functions, especially considering some lncRNAs are expressed both in the nucleus and cytoplasm.

## LncRNAs in angiogenesis and vascular diseases

Many lncRNAs have been studied in the context of angiogenesis and vascular diseases. Please refer to these reviews for recent summaries on this topic [33, 36]. A small number of them have been studied using genetic animal models. By knocking out a short list of lncRNAs in mice, the majority of the lncRNAs are dispensable for embryonic development, suggesting the majority of lncRNAs may act to fine-tune biological processes rather than as master regulators of development [37, 38]. Several notable lncRNAs with genetic studies include *MALAT1*, *MIAT*, and *Leene*. *MALAT1*^*−/−*^ mice showed reduced retinal vascularization, and *MALAT1* deficiency in the *ApoE*^−/−^ background increased atherosclerotic lesion formation in mice [39–41]. LncRNAs *Miat*^−/−^ and *Leene*^−/−^ mice are viable without overt abnormalities. However, *Miat*^−/−^ mice show attenuated pathological hypertrophy and heart failure [42], while diabetic mice deficient in *Leene* demonstrated impaired angiogenesis and perfusion following hind limb ischemia, which can be rescued by *Leene* overexpression [43]. In this paper, we will focus on the lncRNAs that are involved in ocular angiogenesis and vascular diseases in the eye.

## Literature search of lncRNAs involved in vascular oculopathies

Searches of PubMed were conducted for key words including lncRNA, ocular angiogenesis, AMD, diabetic retinopathy, KNV, and ROP. The date range was not filtered since lncRNAs are new discoveries and most of the papers were published within the last 10 years. Review papers were used to further identify the more significant lncRNAs. The lncRNAs were classified as in vivo and in vitro, with more emphasis placed on in vivo, which were then classified into pro-angiogenic and anti-angiogenic lncRNAs. Regulation and function studies of lncRNAs in vascular oculopathies are summarized as below.

## Regulation of LncRNAs in vascular oculopathies

LncRNAs have been shown to be regulated in models of ocular angiogenesis. In a laser-induced choroidal neovascularization (CNV) model, which serves as an animal model for severe wet AMD, microarray analysis revealed significant regulation of 716 lncRNAs, with 442 upregulated and 274 downregulated [44]. *AK036888* topped the list of upregulated lncRNAs, while *ENSMUST00000135495* topped the list of downregulated lncRNAs. A quantitative reverse transcription (qRT)-PCR further confirmed a randomly selected list of lncRNAs. Consistent with the microarray analyses, the expression of lncRNAs *AK148935*, *ENSMUST0000013*, *uc009ewo.1* and *uc029sdr.1* were significantly upregulated and expression of lncRNAs *AK030988*, *ENSMUST00000180519* and *uc007mds*.1 were significantly downregulated in CNV compared to control. Pathway analysis of the lncRNA-mRNA co-expression network indicated that the lncRNA-interacted mRNAs are enriched in the immune system process and chemokine signaling pathway, suggesting that the altered lncRNAs are involved in immunological regulation. Similarly, in an independent study focusing on the RPE-choroid-sclera complexes of CNV mice, 128 lncRNAs were significantly altered [45]. LncRNA *H19*, *NEAT1* and *XLOC_028350* were the top three up-regulated lncRNAs with significant differences. In a mouse model of oxygen-induced retinopathy (OIR), 198 lncRNAs were upregulated and 175 were downregulated [46]. The expression of four lncRNAs including *ENSMUST00000165968*, *ENSMUST00000153785*, *ENSMUST00000134409*, and *ENSMUST00000154285*, were validated, and their gene expression changes correlated with their nearby genes. Gene Ontology (GO) and Kyoto Encyclopedia of Genes and Genomes (KEGG) analyses indicated that the lncRNA-interacted mRNAs were enriched in blood vessel development, angiogenesis, cell adhesion molecules and leukocyte transendothelial migration pathways. Furthermore, in a study using high-throughput sequencing, 57 lncRNAs were differentially expressed in the retinal tissue in a mouse OIR model, with 43 upregulated and 14 downregulated [47]. The differentially expressed genes may regulate ROP in mice via microRNA and multiple signaling pathways. In a streptozotocin (STZ)-induced diabetes model, about 303 lncRNAs were aberrantly expressed in the retinas of early DR, including 89 upregulated and 214 downregulated lncRNAs [48]. Among them, *KCNQ1OT1* is upregulated in aqueous humor and serum samples of DR patients compared to normal subjects, as well as in human retinal ECs (hRECs) cultured in high glucose (HG) conditions [49]. *HEIH* is highly expressed both in the serum of diabetic subjects with DR and in ARPE-19 cells treated with HG conditions [50, 51]. Additionally, blood samples from DR patients and hRECs induced by HG display increased levels of the FOXF1 adjacent Non-coding Developmental Regulatory RNA (*FENDRR*), also known as *FOXF1-AS1*, paralleled with high expression of VEGF [52]. Based on these studies, a panel of lncRNAs were tested in the serum of DR patients to see if they could be a diagnostic and prognostic tool for DR [53]. Significant increased expression of lncRNAs *ANRIL*, *H19*, *HOTAIR*, *HULC*, *MIAT*, *WISPER* and *ZFAS1* were observed in the serum of diabetic patients (with varying stages of DR) compared to non-diabetics. However, there were no notable associations found between lncRNA expression and levels of creatinine or glycated hemoglobin (HbA1C). Additionally, several lncRNAs have shown reduced expression in DR, including *MEG3* and *H19*. *LUADT1* exhibits downregulation solely in T2D patients with DR, implying that the reduced expression of this lncRNA in DR may be linked with retina lesions rather than with hyperglycemia [54]. For other lncRNAs in DR, refer to the review paper [55].

## Functional study of lncRNAs in ocular angiogenesis

Here we summarize the individual lncRNAs that are involved in ocular angiogenesis, with more emphasis on those with in vivo functional studies. They are classified into pro-angiogenic and anti-angiogenic lncRNAs.

### Proangiogenic lncRNAs (Table 2)

**Table 2:** Pro-angiogenic lncRNAs

lncRNAs	Regulation in Disease Models	Function	Mechanism	Refs
ANRIL	Upregulated in HG-treated hRECs, retinas of DR rats and the serum and vitreous of DR patients	Contribute to pathological DR vascular phenotypes and inflammation	Promote NF-kB pathway and enhances VEGF expression through interacting with p300 and EZH2, which regulates miR200b	[53, 56, 58, 63]
H19	Up- or down-regulated in Diabetics or DR, upregulated in corneal neovascularization	proangiogenic but could inhibit endMT and inflammation	Inhibits endMT by inhibiting TGF-β1 and MAPK-ERK1/2 pathway; inhibit inflammation by sponging miR-19b that targets SIRT1; enhances angiogenesis by sponging miR-29c that targets VEGF-A	[71, 73, 76–78]
HDAC4-AS1	Increased in hypoxic ARPE-19 cells	unknown	Enhances HDAC4 transcription activity by recruiting HIF-1α to the HDAC4 promoter	[128]
HIF1A-AS2	Upregulate by hypoxia in ECs, and in the serum of diabetic and proliferative DR patients	Enhance EC angiogenic activities	Acts as ceRNA for miR-153-3p that targets HIF-1α	[127]
HOTAIR	upregulated in HG-treated hRECs, in the retina of DR mice and the vitreous humor and serum from proliferative DR patients	Contribute to diabetic vascular phenotypes	Binds to PRC2 and LSD, which regulates VEGF-A, ANGPTL4, PFGF and HIF1α transcription.	[64–67]
HOTTIP	Increased in STZ-induced DR mice and rats	Contribute to diabetes-induced visual function decline and retinal inflammation	Activates p38/MAPK pathway, and promotes angiogenesis by interacting with WDR5 and MLL, which regulates ICAM-1 and VEGF transcription	[70, 139–141]
IPW	Upregulated in hypoxic ECs and the choroid of laser induced CNV in mice	Promote choroidal sprouting angiogenesis and laser-induced CNV	Represses miR-370 at the DLK1-DIO3 locus, which targets KDR and BMP-2	[82]
lncEGFL7OS		Enhances human angiogenesis	Interacts with MAX protein via to regulate EGFL7/miR-126 expression, therefore promoting MAPK and AKT pathway	[134]
MALAT1	Upregulated in HG-treated hRECs, OIR mouse model and diabetic patients	Promotes retinal vascularization, and contributes to DR vascular phenotypes	Acts as ceRNA for miR203a-3p, miR-205-5p, miR-124-3p, and miR-320 that target VEGF, HIF1 and EGR1, and promotes inflammation by elevating IL-1β, IL-6 and TNF-α levels.	[39, 48, 85–91]
MIAT	Upregulated by HG in cell lines, in diabetic rats and mice and in the plasma of DR patients	Contributes to diabetic-induced vascular phenotypes	Acts as ceRNA for mirR-150-5p that targets VEGF	[93–96]
NEAT1	Up- or down-regulated by HG-treated retinal cells, downregulated in STZ-treated mice but upregulated in the serum of DR patients	Promotes laser-induced CNV	Enhances angiogenesis by acting as ceRNA for miR-19-5p that targets TGB-βR2 and therefore enhancing VEGF-A expression; Inhibits resolving inflammation by inhibiting M2 macrophage polarization by acting ceRNA for miRNA-148a-3p that targets PTEN.	[100–102]
SNHG16	Upregulated in HG-treated ECs and the plasma of proliferative DR patients	Promotes angiogenesis in ECs but attenuates oxidative stress-induced angiogenesis	Acts ceRNA for multiple miRNAs	[123–126]
TUG1		Enhances retinal neovascularization and inflammation in mouse OIR model	Enhances angiogenesis by acting as ceRNA for miR-34a-5p and miR-299 that targets VEGF, miR-204-5p that targets JAK2, miR-145-5p that targets CCN1 and miR-29c-3p that targets PDGF-BB. It also promotes inflammation by elevating TNF-α, IL-1β and IL-6	[110, 111]
Vax2os1 and Vax2os2	Upregulated in the aqueous humour of wet AMD patients, and dynamically regulated in OIR model		Interacts with C1D and PATL2	[131, 132]

#### ANRIL

**Fig. 3:**
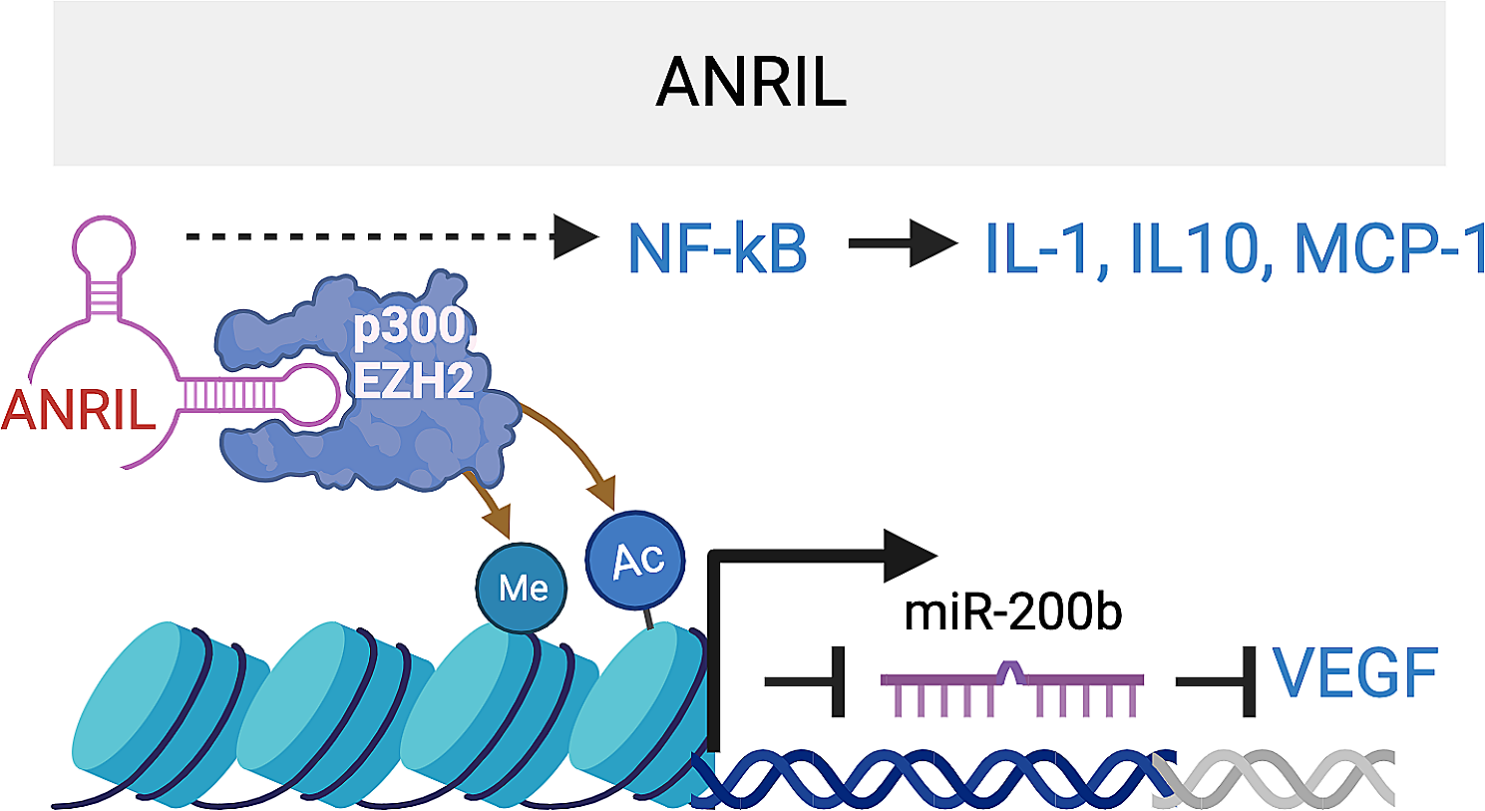
ANRIL lncRNA Promotes inflammation by regulating NF-kB pathway and enhances VEGF expression and angiogenesis through interacting with p300 and EZH2, which regulates miR200b. The target sequence in VEGFA that is complementary to miR200b seed region is CAGUAUU (1314–1320) in VEGFA 3’UTR

Antisense RNA to INK4 locus (ANRIL) is a significant lncRNA in cardiovascular diseases, diabetes, and cancer. ANRIL’s single nucleotide polymorphism associates with type II diabetes and coronary artery diseases [56]. It is detectable in human and mouse RECs and upregulated under HG conditions [57]. Also, it’s elevated in the retina of STZ-induced DR rats and in the serum and vitreous of DR patients [53, 58]. *ANRIL* silencing using siRNAs prevented HG-induced VEGF expression and vascular tube formation in hRECs, and *ANRIL*^*−/−*^ mice are phenotypically normal but are protected from diabetes-induced VEGF upregulation [59]. Conversely, overexpression of *ANRIL* upregulates VEGF and promotes angiogenesis [60]. Mechanistically, *ANRIL* binds to both p300 and EZH2 of the Polycomb Repressive Complex (PRC) 2 to regulate VEGF expression. The upregulation of miR200b in si-*ANRIL*-transfected hRECs suggests the involvement of mir200b in the ANRIL-regulated VEGF expression. Combined with the data that VEGF is a target gene of miR-200b, and PRC2 represses miR-200b, ANRIL likely regulates VEGF expression by interacting with p300 and EZH2 in the PRC2 complex, which in turn represses miR-200b expression (Fig. 3) [61, 62]. In an independent study, the infection of si-*ANRIL* into STZ-induced DR rats reversed DR pathologies [58]. Downregulation of the NF-k pathway by si-*ANRIL* in the retina was confirmed by reduced P65 phosphorylation and the mRNA level of inflammatory markers (IL-1, IL-10, and MCP-1). In another relevant study, adenovirus mediated *ANRIL* overexpression increased Akt and eNOS [63]. These establish ANRIL as a key pro-angiogenic and pro-inflammatory lncRNA with implications in DR (Fig. 3).

#### HOTAIR

**Fig. 4:**
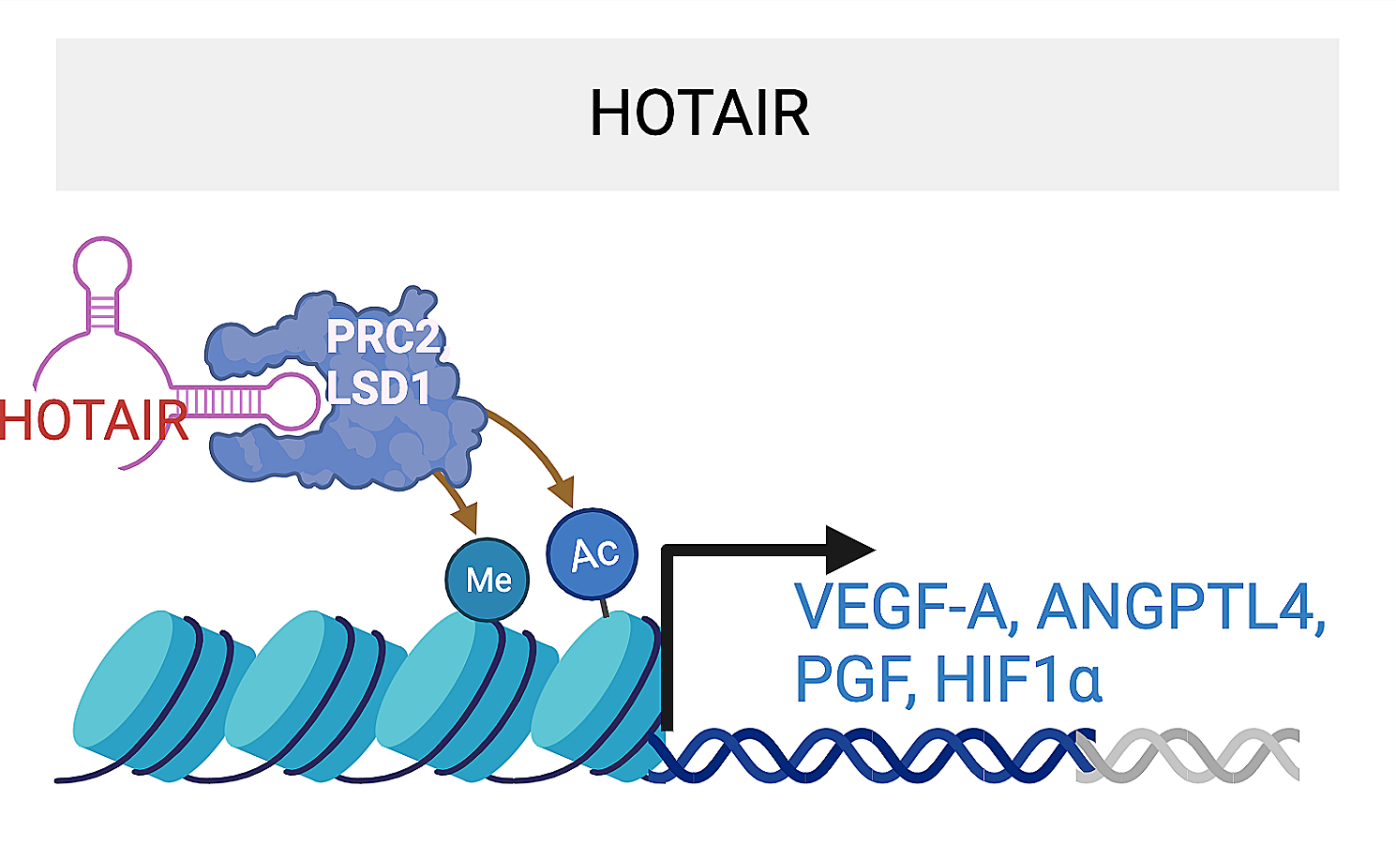
HOTAIR lncRNA promotes angiogenesis by binding to PRC2 and LSD, which regulates VEGF-A, ANGPTL4, PFGF and HIF1α transcription

*HOX antisense intergenic RNA* (*HOTAIR*) was initially identified for its role in cancer progression and metastasis [64, 65]. It is highly conserved in mouse, rat and human. Its expression is upregulated in human RECs under HG condition, in the retina of STZ-induced DR mice, and in the vitreous humor and serum from proliferative DR patients [66, 67]. *HOTAIR* knockdown repressed REC proliferation and migration, and vessel-like structure formation under HG condition *in vitro.* Delivering of *HOTAIR* shRNA by AAV virus or *HOTAIR* siRNA alleviated retinal vessel impairment in STZ-induced DR mice and rats, as shown by decreased acellular capillaries, increased pericytes in the retina, and decreased retinal vascular leakage. Mechanistically, *HOTAIR* silencing represses the expression of multiple RNA transcripts implicated in angiogenesis, such as *VEGFA*, *ANGPTL4*, *PGF* (placental growth factor), and *HIF1*α. *HOTAIR* has been shown to regulate target gene transcription by binding to histone methylase PRC2 and histone demethylase LSD1 [68]. *HOTAIR* also binds to histone demethylase LSD1 and HIF1, or recruits RNA polymerase II and histone methyltrasferase EZH2 and acetylators (P300) to VEGF promoter and enhance its transcription. HOTAIR also binds to LSD1 to inhibit the H3K4me3 on the VE-cadherin promoter, thereby suppressing VE-cadherin transcription. Therefore, *HOTAIR* is a proangiogenic lncRNA with potential to regulate angiogenesis and vascular permeability in DR. Overall, *HOTAIR* functions as a scaffold for transcription factors that increase expression of pro-angiogenic genes (Fig. 4).

#### HOTTIP

**Fig. 5:**
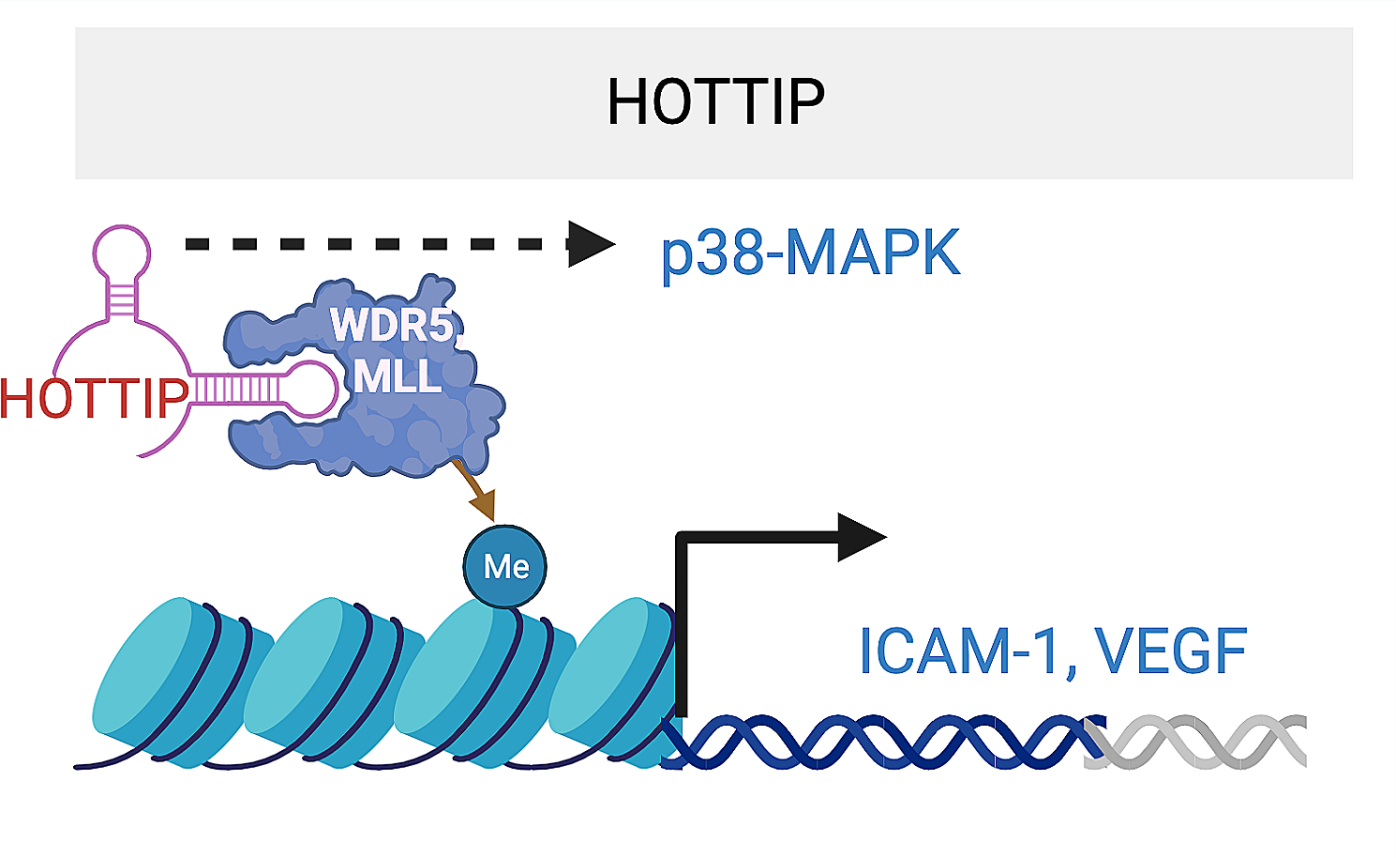
HOTTIP lncRNA promotes inflammation and angiogenesis by activating p38/MAPK pathway, as well as by interacting with WDR5 and MLL, which regulates ICAM-1 and VEGF transcription

(HOXA transcript at the distal tip) (*HOTTIP*), located as the distal 5’ tip of the HOXA locus, is a critical oncogenic lncRNA [69]. It functions as an important mediator of chromatin activation by recruiting WD repeat-containing protein (WDR5) and mixed lineage leukemia (MLL) (Fig. 5). *HOTTIP* expression is significantly upregulated in STZ-induced DR mice and rats. Adenovirus-delivered shRNA against *HOTTIP* alleviated diabetes-induced visual function decline and apoptosis of retinal cells and reduced the expression of ICAM-1 and VEGF in the retina. Mechanistically, it functions by activating the p38-MAPK pathway in retinal ECs [70]. Its underlying mechanism in DR is yet to be discovered.

#### H19

**Fig. 6:**
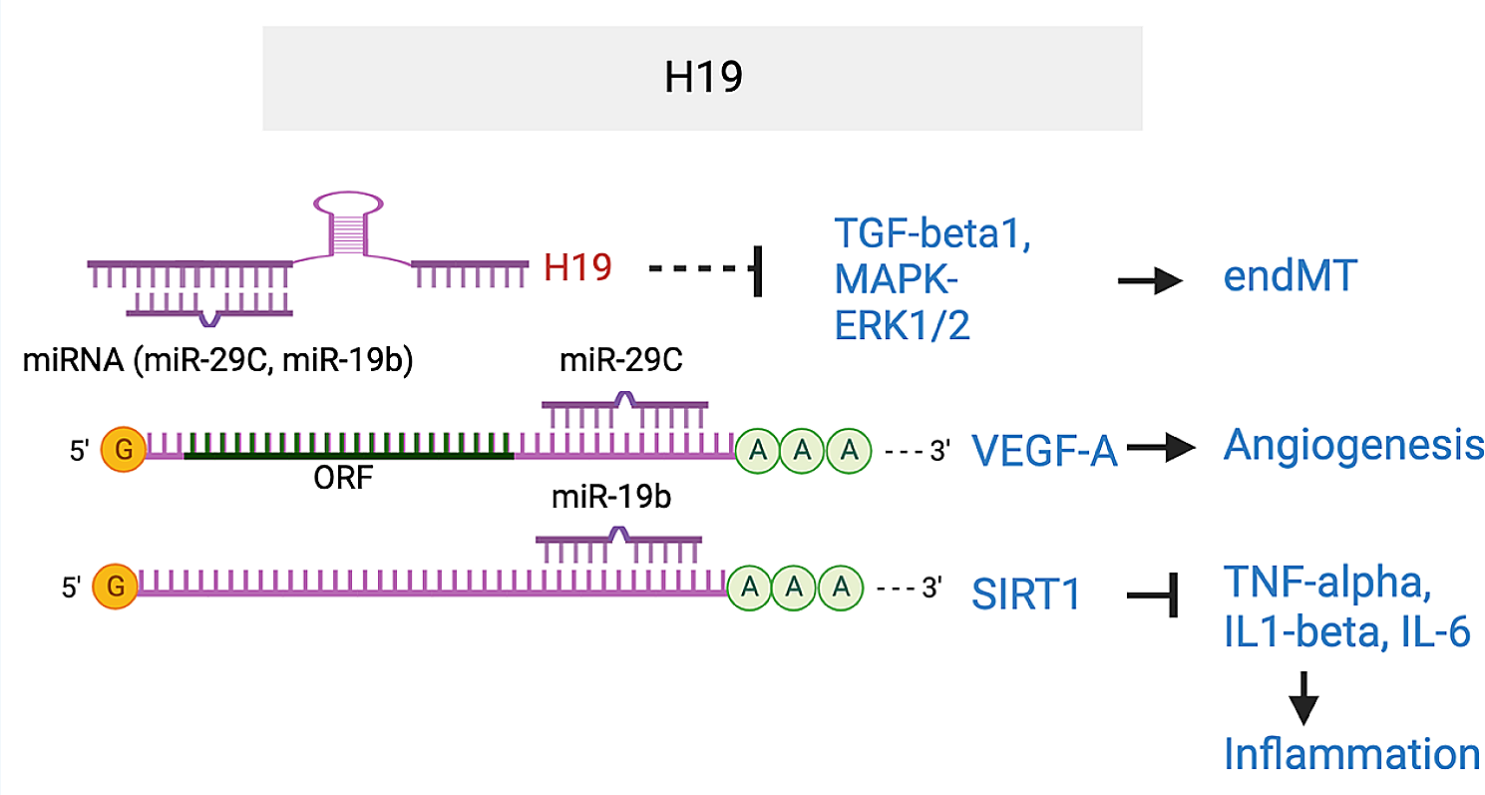
H19 lncRNA Inhibits endMT by inhibiting TGF-β1 and MAPK-ERK1/2 pathway; inhibits inflammation by sponging miR-19b that targets SIRT1; enhances angiogenesis by sponging miR-29c that targets VEGF-A. The target sequence in SIRT1 that is complementary to miR-19-3p seed region is UUGCAC (1285–1291) in SIRT1 3’UTR. The target sequence in VEGFA that is complementary to miR-29c seed region is UGGUGCUA (1758–1765) in VEGFA 3’UTR

*H19*, one of the early discovered lncRNAs, is maternally expressed in the *H19-Igf2* locus and overexpressed in multiple cancers [71, 72]. A differentially methylated H19 upstream region determines the reciprocal expression of H19 from the maternal allele and Igf2 from the paternal allele. As a lncRNA, *H19* also serves as a source of miR-675, which restricts Igf1r expression. Additionally, *H19* acts as ceRNA for multiple miRNAs, being linked to multiple pathological processes, including inflammation, angiogenesis, neurogenesis, and fibrosis progression [71, 73, 74]. Regarding ocular angiogenesis, *H19* is markedly upregulated in the vascularized corneal and can be induced by bFGF in ECs. It promotes EC angiogenesis by sponging miR-29c that targets VEGFA [75]. In the context of DR, downregulation of *H19* was observed in vitreous humor samples from individuals with DR, in the retina of diabetic mouse models and in HG-induced hREC and ARPE-19 cells [76–78]. Conversely, Fawzy et al. observed *H19* upregulation in the plasma of diabetic patients compared to healthy subjects, with no significant differences between patients with and without DR [79]. *H19* overexpression prevented HG-induced endothelial–mesenchymal transition (endMT), while its silencing led to endMT phenotypes similar to HG induction. In *H19*^*−/−*^ mice, endMT phenotypes, shown by reduced *Cd31* and *Ve-Cad* expression, increased *Fsp1* and *α-SMA* expression, and extravascular IgG staining, were also observed. Mechanistically, *H19* represses endMT by suppressing TGF-β1 and by repressing the MAPK–ERK1/2 signaling pathway. Beyond endMT, *H19* also inhibits HG-induced expression of inflammatory cytokines, such as *TNF-α*, *IL-1β* and *IL-6* in ARPE-19 cells, by sponging miR-19b which targets silence information regulator factor related enzymes 1 (SIRT1). These studies suggest *H19* is a proangiogenic lncRNA but can inhibit multiple pathological processes, including endMT and inflammation, in DR models (Fig. 6). Further in vivo studies are needed to test the role of *H19* in ocular angiogenesis, especially proliferative DR or neovascular AMD.

#### IPW

**Fig. 7:**
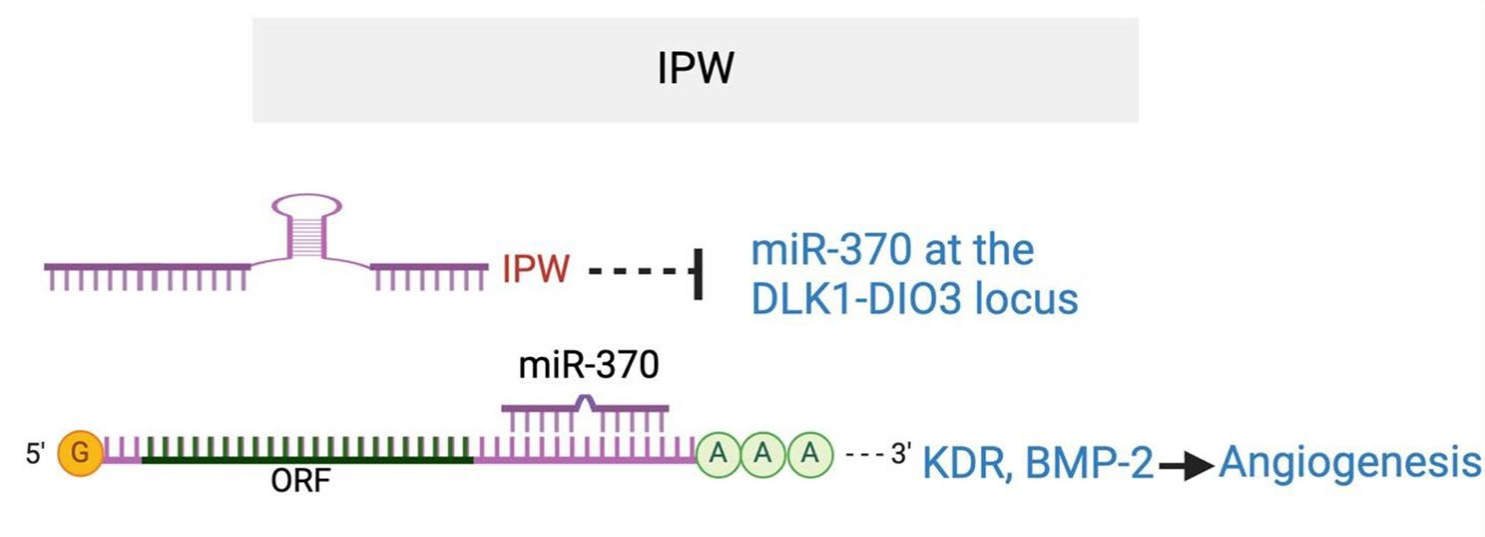
IPW lncRNA enhances angiogenesis by repressing miR-370 at the DLK1-DIO3 locus, which targets KDR and BMP-2. The target sequence in KDR that is complementary to miR-370 seed region is CAGCAGG (79–86) in KDR 3’UTR. The target sequence in BMP-2 that is complementary to miR-370 seed region is AGCAGG (725–731) in BMP-2 3’UTR

*IPW (Imprinted gene in the Prader-Willi syndrome (PWS) region)* was initially identified in an epigenetic PWS disorder [80]. Located in the critical region of the PWS locus, *IPW* regulates the imprinted DLK1-DIO3 region [81]. Disruption of the *IPW* region is associated with neurogenic disorders in humans. IPW was found to be significantly up-regulated in the choroids in laser-induced CNV mouse model and in hypoxic ECs [82]. *IPW* silencing or overexpression reduced or increased EC viability, proliferation, migration, and tube formation in vitro, as well as choroid sprouting angiogenesis *ex vivo. IPW* silencing also inhibited laser-induced CNV in vivo. Mechanistically, *IPW* silencing caused upregulation of miR-370 but not the miR-409 and *MEG3* genes in the DLK1-DIO3 locus. miR-370 has been shown to inhibit angiogenesis by targeting KDR and BMP-2 [82, 83]. *IPW* overexpression rescued the anti-angiogenic effect of miR-370 in the ex vivo sprouting angiogenesis model and in vivo laser-induced CNV model. These suggest *IPW* as a pro-angiogenic lncRNA that functions by repressing miR-370, and *IPW* silencing could be a promising strategy for treating neovascular ocular diseases (Fig. 7).

#### MALAT1

**Fig. 8:**
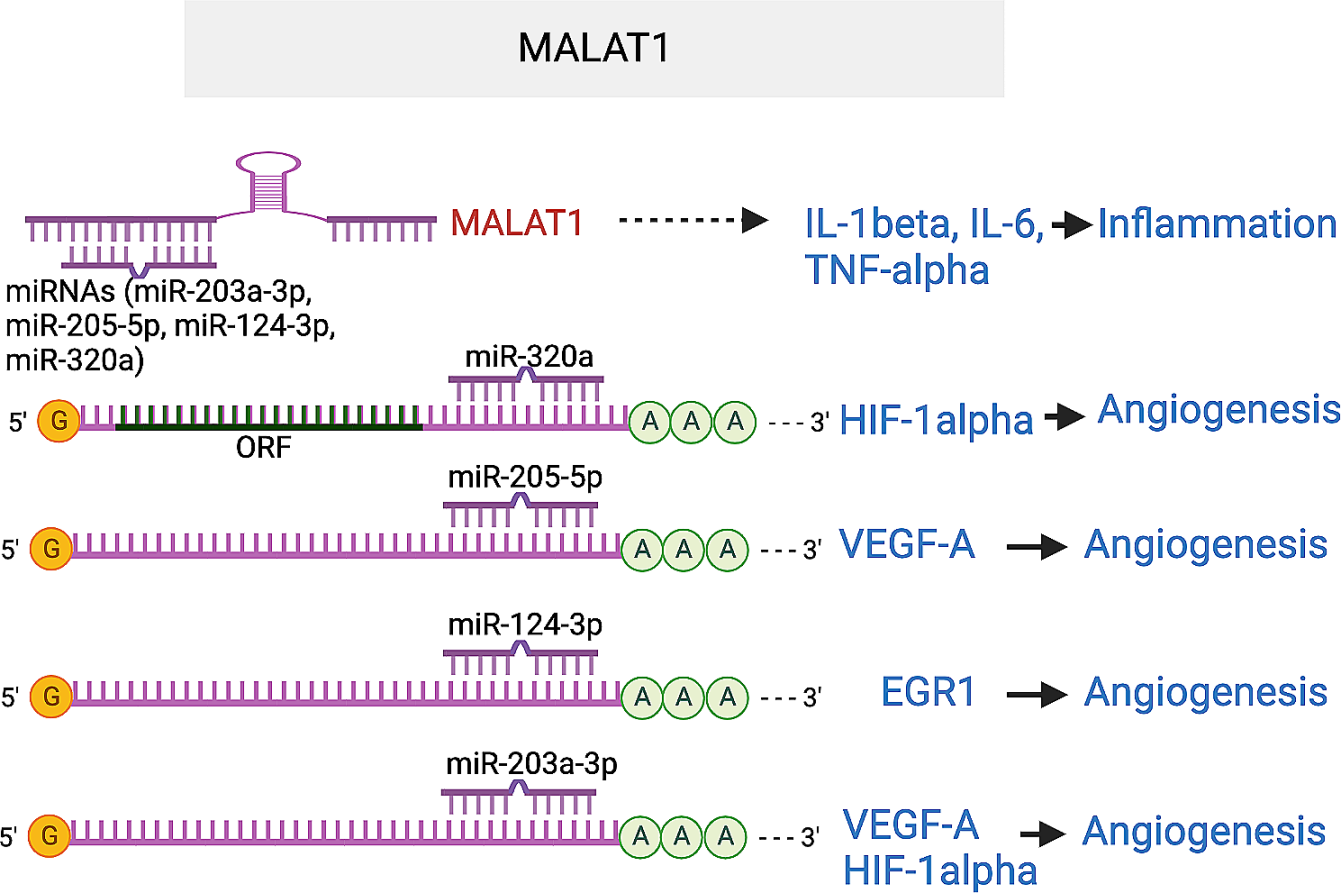
MALAT1 lncRNA promotes angiogenesis by acting as ceRNA for miR203a-3p, miR-205-5p, miR-124-3p, and miR-320 that target VEGF, HIF1α and EGR1, and promotes inflammation by elevating IL-1β, IL-6 and TNF-α levels. The target sequences in HIF1α that is complementary to miR-320a and miR-203a seed region are AGCUUU (1846–1852) and AUUUCA (995–1001) in HIF1α 3’UTR. The target sequences in VEGFA that is complementary to miR-205-5p and miR-203-3p seed regions are AUGAAGG (167–173) and AUUUCA (1320–1326) in VEGFA 3’UTR. The target sequence in EGR1 that is complementary to miR-124-3p seed region is GUGCCUU (769–775) in EGR1 3’UTR

*Metastasis-associated lung adenocarcinoma transcript 1* (*MALAT1*) was initially discovered as a predictor for lung cancer metastasis [84]. In an OIR murine model and HG-stimulated hRECs, *MALAT1* was upregulated together with VEGF and HIF1a, while miR-203a-3p was downregulated. *MALAT1* was also upregulated in the aqueous humor samples, and in fibrovascular membranes of diabetic patients [48]. Knockdown of *MALAT1* blunted HG-induced proliferation, migration, and tube-like structure formation in human retinal microvascular ECs (hRMEC), mouse RMEC and HUVEC cells [39, 85–88]. Mechanistically, *MALAT1* was found by several studies to function as ceRNA to sponge multiple miRNAs, including miR-203a-3p, miR-200b, miR-205-5p, miR-124-3p and miR-320a, which relieve the repression of angiogenic genes VEGF, HIF1 and EGR1 by these miRNAs. Genetic ablation of *MALAT1* reduced EC proliferation and neonatal retina vascularization [89]. GapmeRs-induced pharmacological inhibition of *MALAT1* led to decreased blood flow recovery and capillary density in a hindlimb ischemia model. In STZ-induced diabetic rats, *MALAT1* knockdown by shRNA injection ameliorated DR phenotypes in vivo, including pericyte loss, capillary degeneration, microvascular leakage, and retinal inflammation [39]. In addition, inhibiting retinal *MALAT1* alleviated retinal neurodegeneration in the STZ mouse model [90]. In the OIR mouse model, silencing of *MALAT1* by intravitreal siRNA injection inhibited retinal neovascularization in vivo, shown by reduced neovascular tufts, non-perfusion region, and EC nuclear counts [91]. The expression of CCN1, phosphor-AKT, and VEGF, which was upregulated by hyperoxia in the retina, was shown to be downregulated by si-*MALAT1*. In addition, the expression of inflammatory cytokines IL-1β, IL-6, and TNF- α were reduced by *MALAT1* silencing. These suggest that *MALAT1* could sponge multiple miRNAs to promote angiogenesis and potentially inflammation. Consequently, *MALAT1* may serve as a potential therapeutic target for DR and ROP (Fig. 8).

#### MIAT

**Fig. 9:**
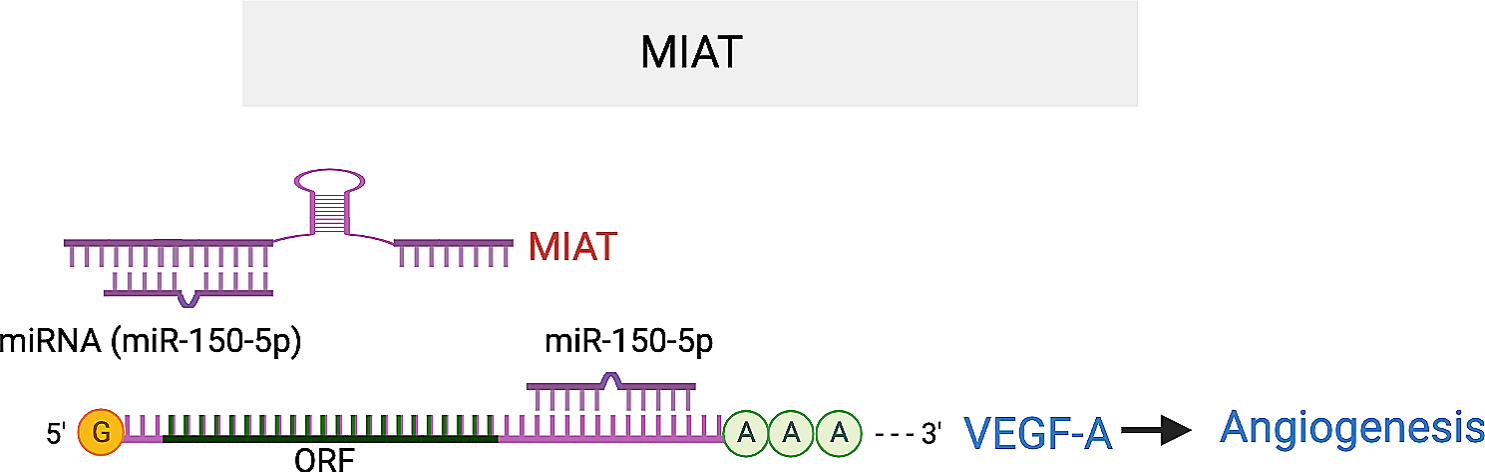
MIAT lncRNA promotes angiogenesis by acting as ceRNA for mirR-150-5p that targets VEGF. The target sequences in VEGFA that is complementary to miR-150-5p seed region are UUGGGA (676–681) and GGGGA (789–794) in VEGFA 3’UTR

*MIAT* was initially discovered as a myocardial infarction (MI)-associated transcript (*MIAT*) [92]. Its expression was shown to be upregulated in the plasma of DR patients compared to both patients without DR and healthy individuals [93]. It is also upregulated by HG in different cell lines, and in the retina of diabetic rats and STZ-treated mice [94–96]. *MIAT* knockdown significantly reduced EC proliferation but increased EC cell death under HG. It also inhibited EC migration and tube-like structure formation induced by TNF-α or VEGF. Silencing *MIAT* in STZ-induced diabetic rats alleviated diabetic-induced retinal neovascularization, vascular leakage, and inflammation in vivo. Mechanistically, *MIAT* enhances the expression of VEGF by acting as a miR-150-5p sponge. Under hyperglycemia or hypoxic stress that induces angiogenesis, *MIAT* is upregulated, relieving the miR-150-5p associated repression of VEGF [94]. Consistently, endothelial miR-150 has been shown to be an intrinsic suppressor of pathological ocular neovascularization [97]. These findings established *MIAT* as an endogenous miR-150-5p sponge, driving the stimulation of ocular angiogenesis (Fig. 9).

#### NEAT1

**Fig. 10:**
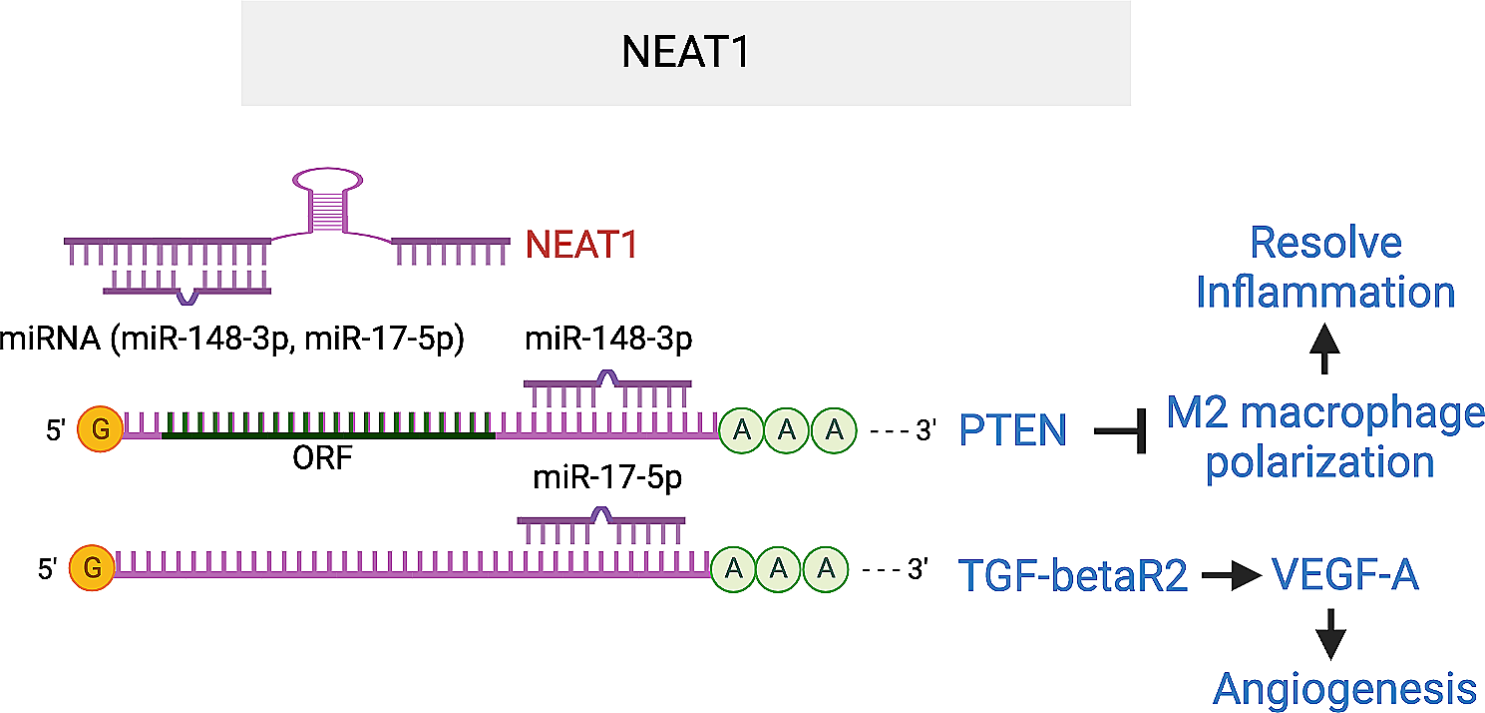
NEAT1 lncRNA enhances angiogenesis by acting as ceRNA for miR-19-5p that targets TGB-βR2 and therefore enhancing VEGF-A expression. It also prevents resolving inflammation by inhibiting M2 macrophage polarization by acting ceRNA for miRNA-148a-3p that targets PTEN. The target sequences in PTEN that is complementary to miR-148-3p seed region are UGCACUG (2254–2260) and UGCACUG (3151–3158) in PTEN 3’UTR. The target sequence in TGF-βR2 that is complementary to miR-17-5p seed region is GCACUUU (268–275) in TGF-βR2 3’UTR

*Nuclear-enriched abundant transcript 1 (NE AT1)*, also known as *nuclear paraspeckle Assembly Transcript 1*, is an abundant, ubiquitously expressed lncRNA, which forms a scaffold for a specific RNA granule in the nucleus, the paraspeckle. It has been implicated in multiple neurodegenerative diseases and other diseases [98, 99]. Contrasting results were observed regarding the expression of *NEAT1* in DR. While downregulated in the retinal of STZ-treated mice, but upregulated in the serum of DR patients, it displays both up- or down-regulation in HG-treated retinal cells [100–102]. *NEAT1* is upregulated in laser-induced CNV and M2 macrophage [45]. *NEAT1* silencing in hRECs ameliorated HG-triggered apoptosis, oxidative stress, and inflammation through inactivating TGF-β1 and VEGF-A. Intravitreal injection of *NEAT1* smart silencer inhibited laser-induced CNV leakage and neovascularization. Mechanistically, *NEAT1* acts as sponge to miRNA-148a-3p to regulate the expression of PTEN, which prevents resolving inflammation by inhibiting M1 to M2 macrophage polarization. In another study, *NEAT1* was shown to promote gastric cancer cell angiogenesis by enhancing proliferation, migration, and tube-like structure formation ability of ECs, by sponging miR-17-5p which targets TGFβR2 directly, which in turn, upregulates angiogenic factors including VEGF-A [103]. Therefore, *NEAT1* lncRNA could promote ocular angiogenesis as a ceRNA for several miRNAs miR-148-3p and miR-17-5p to regulate PTEN and TGFβR2 (Fig. 10).

#### TUG1

**Fig. 11:**
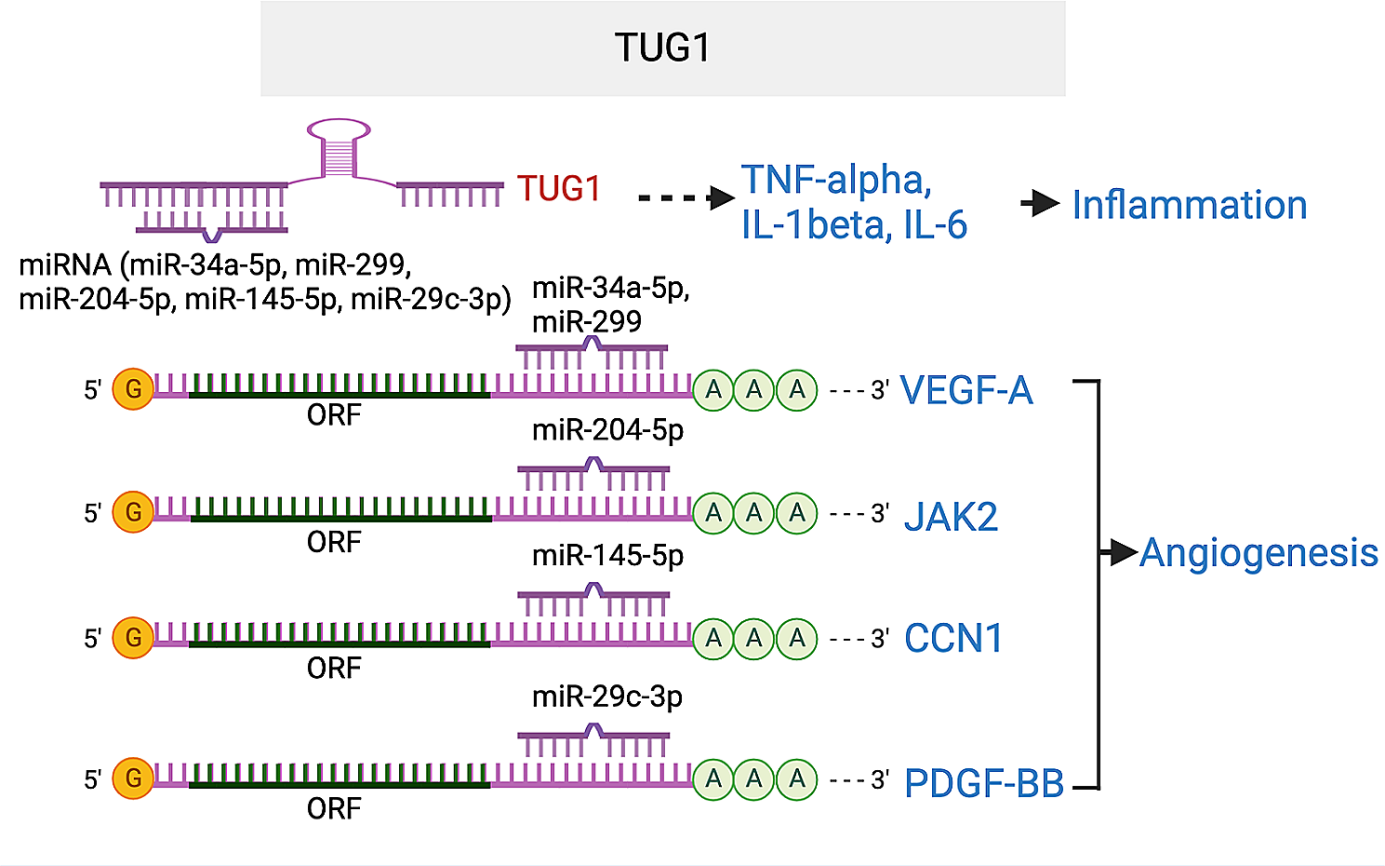
TUG lncRNA enhances angiogenesis by acting as ceRNA for miR-34a-5p and miR-299 that targets VEGF, miR-204-5p that targets JAK2, miR-145-5p that targets CCN1 and miR-29c-3p that targets PDGF-BB. It also promotes inflammation by elevating TNF-α, IL-1β and IL-6. The target sequences in VEGFA that is complementary to miR-34-5p and miR-299-3p seed regions are CACUGCC (859–865) and CCCACAU in VEGFA 3’UTR. The target sequence in JAK2 that is complementary to miR-204-5p seed region is AAGGGA (1453–1459) in JAK2 3’UTR. The target sequence in CCN1 that is complementary to miR-145-5p seed region is ACUGGA (572–577) in CCN1 3’UTR. The target sequence in PDGFB that is complementary to miR-29c-3p seed region is UGGUGCU (132–138) in PDGFB 3’UTR

*Taurine upregulated 1* (*TUG1*) was established as an oncogenic or tumor suppressor lncRNA by different studies [104, 105]. *TUG1* knockdown suppressed tumorigenesis and tumor-induced angiogenesis in mouse hepatoblastoma or glioblastoma xenograft models [106, 107]. Further studies showed that *TUG1* functions a ceRNA for multiple miRNAs, including mitigating the function of miR-34a-5p and miR-299 in repressing VEGFA expression, the function of miR-204-5p in repressing JAK2/STAT3 pathway, the function of miR-145-5p in repressing CCN1 expression, and the function of miR-29c-3p in regulating PDGF-BB/Wnt signaling [106–111]. In a diabetic limb ischemia mouse model, injection of *TUG1* lentivirus stimulated angiogenesis [111]. In the mouse OIR model, intravitreal injection lentivirus expressing *TUG1* shRNA reduced avascular and neovascular areas and reduced inflammation shown by reduced expression of the inflammatory factors IL-1β, IL-6, and TNF-α in the retina. These suggest that *TUG1* silencing could hold a beneficial role in addressing retinal vascular diseases (Fig. 11).

### Anti-angiogenic lncRNAs (Table 3)

**Table 3:** Anti-angiogenic lncRNAs

lncRNAs	Regulation in Disease Models	Function	Mechanism	Refs
MEG3	Downregulated in diabetic and DR serum, STZ-induced diabetic mice, HG-treated RECs	Alleviates retinal vascular dysfunction	Represses angiogenesis by acting as ceRNA for miR-6720-5p that targets CYB5R2, ceRNA for miR-9, and by inhibiting TGF-β and VEGF expression	[114–117]
PKNY	Decreased by hypoxia in RECs and in the RPE/choroid of the laser-induced CNV model	Inhibits choroid sprouting angiogenesis and laser-induced CNV	Represses angiogenesis by interacting with PTBP1, and acting as ceRNA for miR-124 that targets pro-apoptotic BIM gene	[120]

#### MEG3

**Fig. 12:**
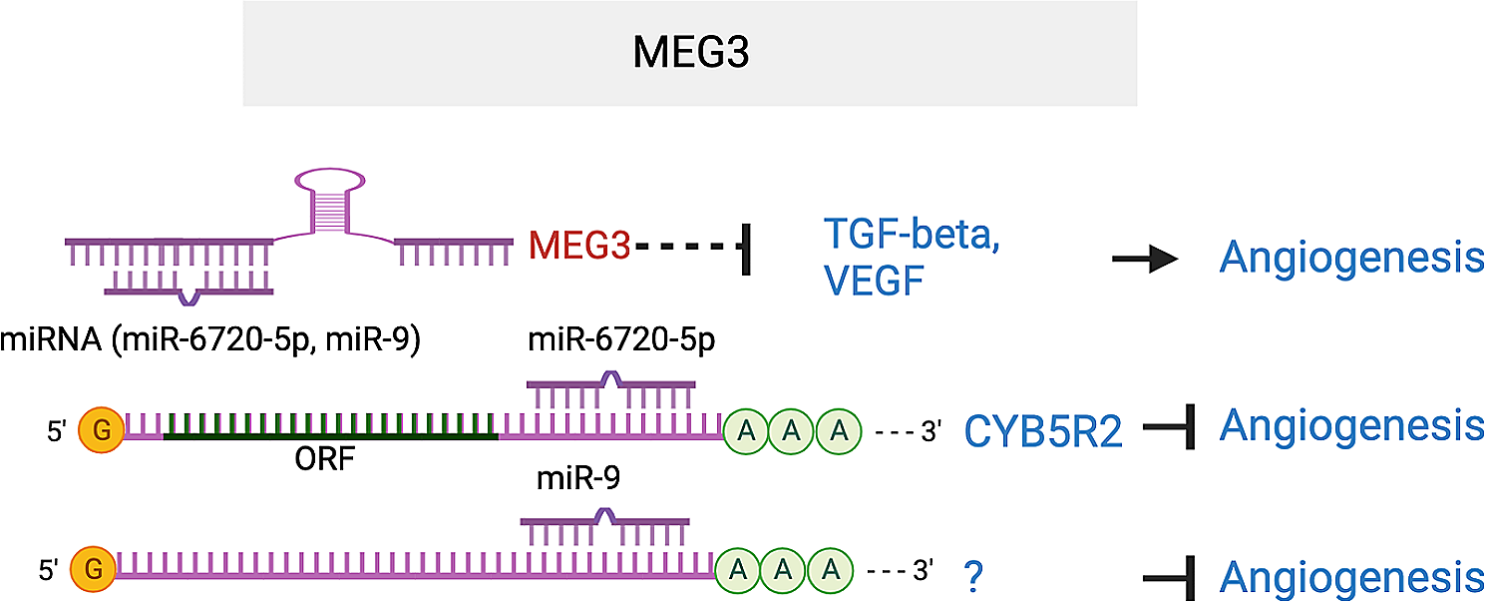
MEG3 lncRNA represses angiogenesis by acting as ceRNA for miR-6720-5p that targets CYB5R2, ceRNA for miR-9, and by inhibiting TGF-β and VEGF expression. The target sequences in CYB5R2 that is complementary to miR-6720-5p seed region are GGCUGGAA (57–64), GGGCUGGA (398–404) and GGCUGGAA (434–441) in CYB5R2 3’UTR

Maternally expressed gene 3 (*MEG3*) is an imprinted lncRNA gene that is expressed in both humans and mice. In many human tumor and tumor cell lines, its expression is lost, indicating that it has a tumor supressive role. *MEG3* has anti-proliferative and pro-apoptotic activities partly through stimulating P53 accumulation [112, 113]. *MEG3* expression is downregulated in the serum of diabetic and DR patients, STZ-induced diabetic mice, and in RECs under HG conditions. *MEG3* overexpression inhibited EC proliferation, survival, and networking, while MEG3 knockdown had the opposite effect, suggesting an anti-angiogenic function of the lncRNA [114]. However, a positive role for MEG3 in VEGF-induced angiogenesis was also reported [115]. Maternal deletion of the *Meg3* gene in mice results in skeletal muscle defects and perinatal death [112]. Upregulation of *VEGFA* gene and genes in VEGF pathways were detected in *Meg3*^−/−^ embryos, consistent with increased cortical microvascular density in the knockout embryos. *MEG3* knockdown by specific shRNAs aggravated retinal vessel dysfunction in vivo, shown by serious capillary degeneration, increased microvascular leakage, and inflammation. Mechanistically, *MEG3* knockdown enhances PI3K/AKT signaling, while *MEG3* overexpression represses high glucose-induced TGF-β and VEGF expression [114, 116]. *MEG3* also acts as a sponge to suppress miR-6720-5p and regulate the expression of cytochrome B5 reductase 2 (CYB5R2), thereby inhibiting neovascularization in DR [117]. In addition, *MEG3* also functions as a ceRNA sponge for miR-9, and miR-9 transfection partially abolished *MEG3*-repressed EC growth and capillary-like structure formation [118]. These studies suggest that *MEG3* is an anti-angiogenic lncRNA by acting as ceRNA for miR-6720-5p and miR-9. *MEG3* elevation may serve as a novel therapeutic strategy for DR (Fig. 12).

#### PKNY

**Fig. 13:**
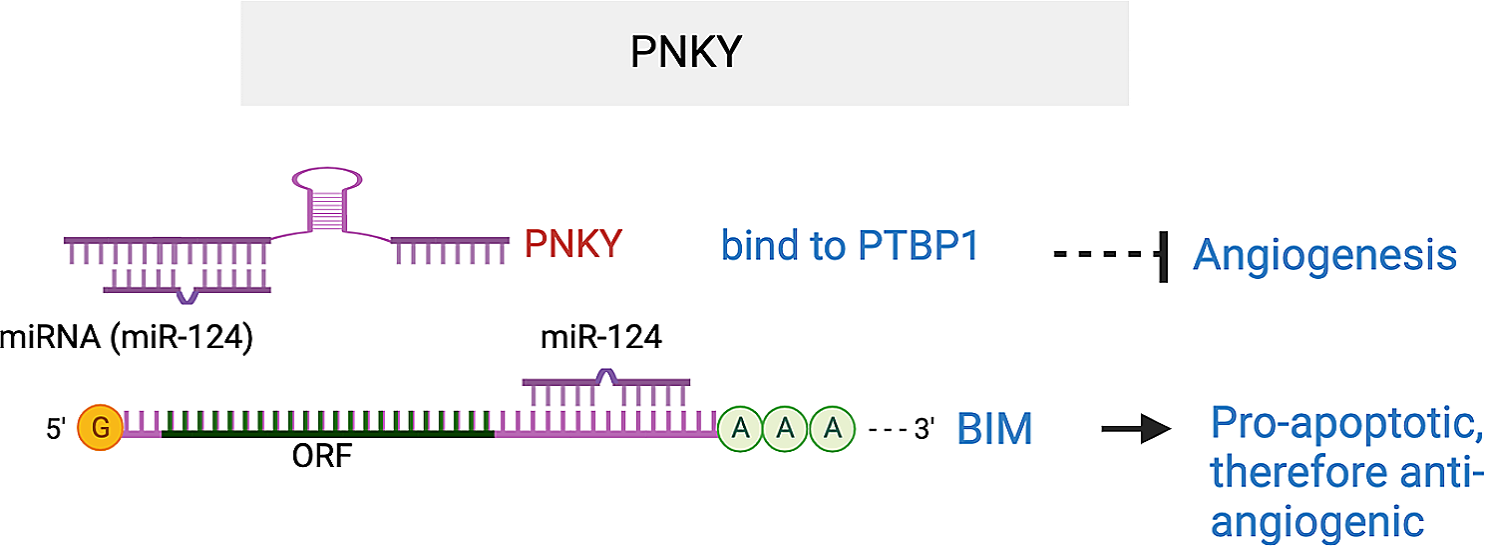
PNKY lncRNA represses angiogenesis by interacting with PTBP1, and acting as ceRNA for miR-124 that targets pro-apoptotic BIM gene. The target sequences in BIM (BCL2L11) that is complementary to miR-124-3p seed region are UGCCUU (465–471) and GUGCCUU (2394–2400) in BIM 3’UTR

*Pinky* (*PKNY*) is an evolutionarily conserved, nuclear enriched lncRNA that regulates neuronal differentiation [119]. Under hypoxia, *PNKY* expression is decreased in RECs and in the RPE/choroid within the context of a laser-induced CNV mouse model [120]. *PKNY* silencing enhanced EC viability, proliferation, migration, and tube-like structure formation in vitro, while *PKNY* overexpression had the opposite effect. Intravitreal injection of AAV virus expressing *PKNY* shRNA led to *PKNY* silencing, resulting in increased choroid sprouting angiogenesis ex vivo and laser-induced CNV *in vivo.* PKNY overexpression by plasmid transfection had the opposite effect. Mechanistically, PNKY interacts with RNA-binding protein polypyrimidine bundle-binding protein 1 (PTBP1) and miR-124, and acts as a sponge for miR-124, therefore upregulating its target protein Bcl-2 like protein 11 (BIM), which is pro-apoptotic. In turn, BIM downregulation causes decreased apoptosis, increased proliferation, and aggravated CNV lesions [120, 121]. . Therefore, PKNY acts as an anti-angiogenic lncRNA that interacts with PTBP1 to regulate miR-124-BIM axis (Fig. 13).

### In vitro studies of lncRNAs

Here we summarize the individual lncRNAs that have the potential to regulate ocular angiogenesis based on in vitro functional studies.

***Small nucleolar RNA host gene (SNHG)16*** is upregulated in human malignant carcinomas. Its expression is elevated in the plasma of patients with proliferative DR, and by HG in hRMECs [122, 123]. Most studies support *SNHG16* promotes proliferation, migration, and vessel-like structure formation in hRMEC and other ECs [122, 123] However, one study suggested that *SNHG16* overexpression attenuated oxidative stress-induced angiogenesis in hMRECs via the miR-195/mfn2 axis [124]. Mechanistically, *SNHG16* functions as ceRNA to sponge miR-20a-5p and miR-101-3p to regulate E2F1 expression, sponge miR-520d-3p to regulate STAT3, or sponge miR-146a-5p and miR-7-5p to regulate interleukin-1 receptor-associated kinase 1 (IRAK1) and insulin receptor substrate 1 (IRS1) and therefore NF-kB and PI3K/AKT pathways [122, 123, 125, 126].

***HIF1A-AS2*** is an antisense transcript to *HIF-1α*). Its expression is upregulated by hypoxia in ECs [127]. *HIF1A-AS2* enhances EC viability, migration, and tube-like structure formation by acting as a ceRNA for miR-153-3p, which regulates HIF-1α/VEGFA/Notch1 cascade by targeting HIF-1α. Its expression is significantly increased in the serum of diabetic patients with the highest levels in proliferative DR patients, which correlates with higher levels of HIFα, VEGF, MAPK, and Endogolin in those patients. Therefore, *HIF1A-AS2* is a pro-angiogenic lncRNA by in vitro studies.

***Histone deacetylase 4 antisense RNA 1*** (***HDAC4-AS1***) is a lncRNA that is increased in hypoxic conditions, and decreased after re-oxygenation in RPE cells, which is inversely correlated with HDAC4 expression [128]. However, in hypoxic conditions, *HDAC4-AS1* knockdown suppressed HDAC4 transcription activity in RPE cells by recruiting HIF-1α to HDAC4 promoter. HDAC4 has been shown to suppress HIF-1α acetylation and enhance HIF-1α transcriptional activity and stability in responding to hypoxia [129, 130]. Therefore HDAC4-AS1 is suggested to be a pro-angiogenic lncRNA, although a functional study is needed.

***Vax2os1*** and ***Vax2os2*** are located in the opposite strand of the Vax2 homeobox transcription factor and were dynamically regulated in the OIR model [131]. These genes are also significantly upregulated in the aqueous humor of patients with neovascular AMD, suggesting a role for these lncRNAs in regulating ocular neovascularization. Mechanistically, *Vax2os1* interacts with C1D while *Vax2os2* interacts with PATL2. Both C1D and PATL2 are important for regulating chromatin structure stability [132].

### Human/primate specific LncRNAs in ocular angiogenesis

As lncRNA genes are less conserved and have less evolutionary pressure compared to protein coding genes, it is not surprising that new lncRNAs would emerge in humans and primates during evolution [133]. Technically, it is challenging to study the function of non-conserved human specific lncRNAs in vivo [28].

**Fig. 14:**
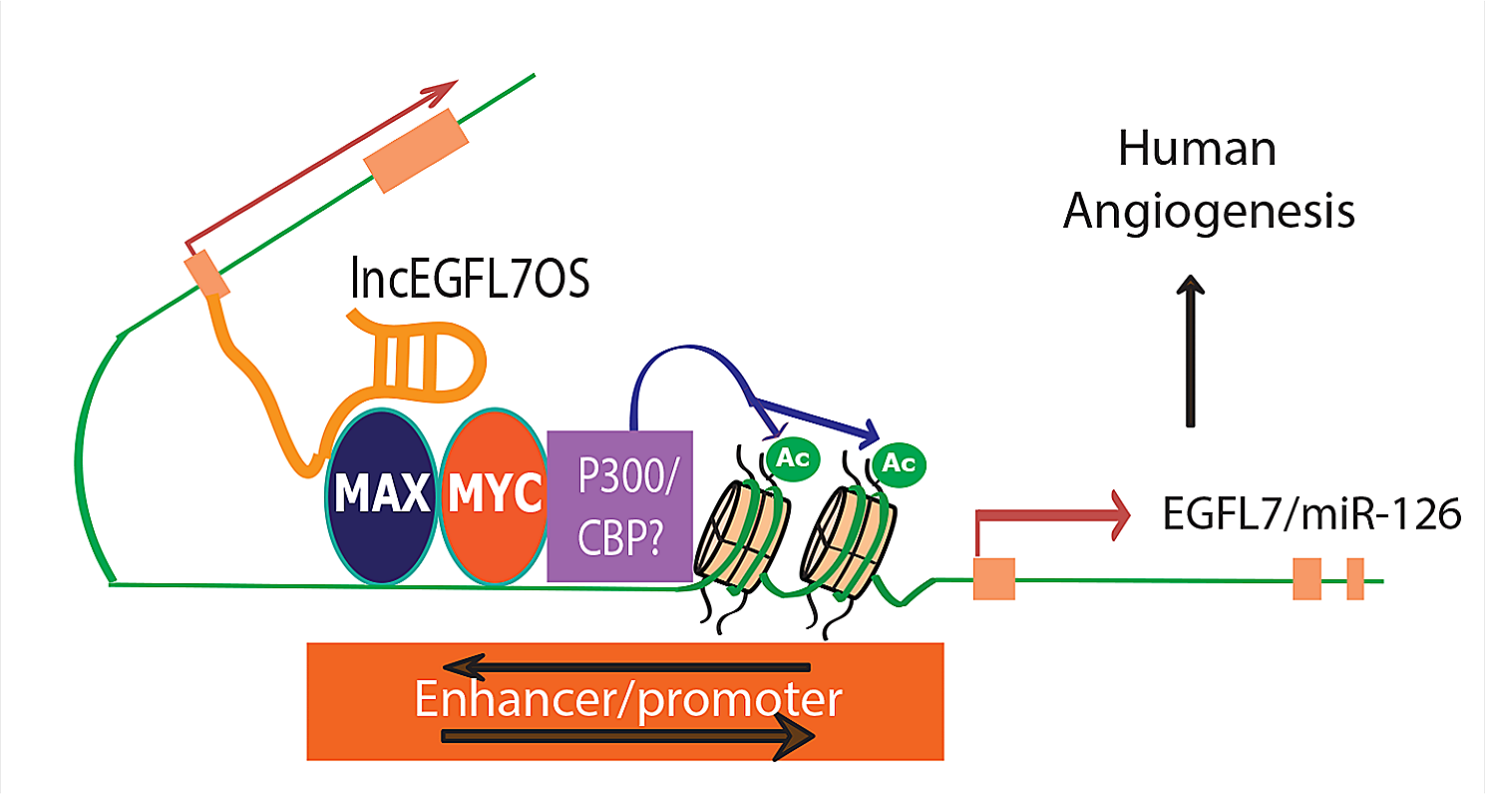
LncEGFL7OS is transcribed in the opposite strand of EGFL7/miR-126 gene under the control of an ETS transcription factors-regulated bidirectional promoter. In turn, lncEGFL7OS transcripts recruit MAX, which interacts with p300 and increase the acetylation of Histone H3K27. This in turn enhances the transcription of EGFL7/miR-126 gene and therefore angiogenesis through MAPK and AKT pathways in human ECs

Although no systematic studies have been reported to characterize human/primate lncRNAs, a few individual lncRNAs have been studied in the context of angiogenesis. By gene expression profiling in several human EC lines, our lab has identified a human/primate-specific EC-enriched *lncEGFL7OS* that is in the opposite strand neighboring the EGFL7/miR-126 gene [134]. Silencing of *lncEGFL7OS* repressed EC proliferation and migration, therefore impairing angiogenesis in vitro, while its overexpression led to enhanced angiogenesis. To directly test the function of *lncEGFL7OS* in ocular angiogenesis in human tissues, an ex vivo human choroid sprouting assay was developed, and *lncEGFL7OS* knockdown by siRNAs drastically repressed human choroid sprouting, establishing a critical role for *lncEGFL7OS* in ocular angiogenesis in human tissues. In line with the reduction of miR-126 by *lncEGFL7OS* silencing, the phosphorylation of ERK1/2 and AKT in reaction to VEGF was inhibited by *lncEGFL7OS* silencing. Mechanistically, MAX knockdown blunted the induction of miR-126 by *lncEGFL7OS* in ECs. MAX transcription factor has been shown to interact with MYC to control cell proliferation and cell death [135]. MYC has been shown to stimulate histone acetylation and gene transcription by recruitment of cAMP-response-element-binding protein (CBP) and p300 [136]. Based on these, the augmentation of H3K27 acetylation by *lncEGFL7OS* likely results from the recruitment of CBP and P300 by MAX/MYC. Taken together, *lncEGFL7OS* acts in cis by interacting with MAX transcription factor to enhance H3K7 acetylation and promote EGFL7/ miR-126 expression (Fig. 14). These suggest that *lncEGFL7OS* is an enhancer-like lncRNA. We have designed CRISPR-mediated targeting of the EGLF7/miR-126/*lncEGFL7OS* locus to inhibit angiogenesis, suggesting therapeutic potential of targeting this locus for vascular diseases, especially vascular retinopathies.

*FLANC* is a human/primate-specific lncRNA that is expressed in normal colon cells but upregulated in colon cancer cells (CRCs) [137]. Modulation of *FLANC* expression influenced cellular growth, apoptosis, migration, angiogenesis, and metastases formation in CRCs and in vivo. In vivo pharmacological targeting of *FLANC* by siRNA carrying nanoparticles reduced angiogenesis and metastases. Mechanistically, *FLANC* induced the overexpression of VEGFA by upregulating and prolonging the half-life of phosphorylated STAT3 Although this is interesting, whether this lncRNA is involved in human/primate ocular angiogenesis warrants further study.

## Conclusion remarks, future directions

Given the fact that an increasing list of lncRNAs are regulated in vascular oculopathies and have shown significant functions in ocular angiogenesis, lncRNAs have garnered considerable attention in the ocular angiogenesis field. While most lncRNAs studied thus far are not necessary for mammalian animal viability, many are highly regulated in retinal vascular disease models and the serum or plasma of patients with vascular oculopathies. In this regard, the expression level of individual lncRNA or a panel of lncRNAs in the serum, plasma or aqueous humor could have prognostic value for vascular oculopathies. Some lncRNAs, such as *ANRIL*, *HOTAIR*, *HOTTIP*, *H19*, *IPW*, *MALAT1*, *MIAT*, NEAT1, and *TUG1*, have been shown to have pro-angiogenic properties, while *MEG3* and *PKNY* have been shown to be anti-angiogenic. Studies on *MALAT1*^−/−^ mice and *Meg3*^*−/−*^ embryos indicate that these lncRNAs play a significant role in vascular development. Several lncRNAs, including *ANRIL*, *H19*, *HOTAIR*, *HOTTIP*, *MALAT1*, and *MIAT1*, have been shown to contribute to diabetic-induced vascular and inflammatory phenotypes. Additionally, *IPW* and *NEAT1* have been shown to promote laser-induced CNV, while *TUG1* has been shown to enhance retinal neovascularization and inflammation in the mouse OIR model. Certain human/primate-specific lncRNAs, such as *lncEGFL7OS*, have been shown to enhance human choroid sprouting angiogenesis. As detailed in the paper, the functional mechanisms of these lncRNAs are diverse, with some acting as ceRNAs and others interacting with proteins to regulate numerous angiogenic factors such as VEGF, HIF1a, and multiple angiogenic/inflammatory signaling pathways including MAPK, PI3K/AKT and NF-kB pathways. Of note, the different functional mechanisms of lncRNAs are not mutually exclusive, as one lncRNA could adopt multiple mechanisms to regulate gene expression and signaling pathways. Based on the current studies, lncRNAs provide an additional and rich layer of gene regulation to due to their diverse mechanisms. These do pose challenges to study the functional mechanisms of lncRNAs. Even for lncRNAs that have been extensively studied, more research is needed to dissect their full functional mechanisms. Current studies already implicated therapeutic potential by targeting lncRNAs. For example, *ANRIL*, *H19*, *HOTAIR*, *HOTTIP*, *MALAT1*, and *MIAT1* could have therapeutic potential in DR, while *NEAT1* and *IPW* could have therapeutic potential in wet AMD. Even for these lncRNAs, more model systems could be used to ascertain their functions in ocular angiogenesis and vascular oculopathies. With the emergence of new technologies for RNA therapeutics, including nanoparticle-conjugated lncRNA overexpression and siRNA-based lncRNA knockdown systems, targeting lncRNA therapeutically could be a reality in the near future. The advantage of RNA-based therapy includes easiness of design based on nucleotide complementarity, quickness and flexibility compared to the lengthy processes of small molecule or antibody development. Ongoing clinical trials targeting lncRNAs for various diseases offer promise for the future [33].

## Data Availability

N/A.

## References

[1] Wong NKP (2020). Pathophysiology of Angiogenesis and its role in Vascular Disease. Mechanisms of vascular disease: a Textbook for Vascular specialists.

[2] Naito H, Iba T, Takakura N (2020). Mechanisms of new blood-vessel formation and proliferative heterogeneity of endothelial cells. Int Immunol.

[3] Raica M, Cimpean AM (2010). Platelet-derived growth factor (PDGF)/PDGF receptors (PDGFR) Axis as Target for Antitumor and Antiangiogenic Therapy. Pharmaceuticals (Basel).

[4] De Falco S, Gigante B, Persico MG (2002). Structure and function of placental growth factor. Trends Cardiovasc Med.

[5] Vimalraj S (2022). A concise review of VEGF, PDGF, FGF, notch, angiopoietin, and HGF signalling in tumor angiogenesis with a focus on alternative approaches and future directions. Int J Biol Macromol.

[6] Fagiani E, Christofori G (2013). Angiopoietins in angiogenesis. Cancer Lett.

[7] Nicholas MP, Mysore N (2021). Corneal neovascularization. Exp Eye Res.

[8] Sharif Z, Sharif W (2019). Corneal neovascularization: updates on pathophysiology, investigations & management. Rom J Ophthalmol.

[9] Dai C et al. Concurrent physiological and pathological angiogenesis in retinopathy of Prematurity and emerging therapies. Int J Mol Sci. 2021;22(9). 10.3390/ijms22094809 PMC8124946 34062733

[10] Wang W, Lo ACY. Diabetic retinopathy: pathophysiology and treatments. Int J Mol Sci. 2018;19(6). 10.3390/ijms19061816 PMC6032159 29925789

[11] Nithianandan H, Sridhar J (2020). Surgical and Medical Perioperative Management of Sickle Cell Retinopathy: A literature review. Int Ophthalmol Clin.

[12] Das A, McGuire PG (2003). Retinal and choroidal angiogenesis: pathophysiology and strategies for inhibition. Prog Retin Eye Res.

[13] Zhao F (2018). Expression, regulation and function of miR-126 in the mouse choroid vasculature. Exp Eye Res.

[14] Nowak JZ (2006). Age-related macular degeneration (AMD): pathogenesis and therapy. Pharmacol Rep.

[15] Brown DM (2006). Ranibizumab versus Verteporfin for neovascular age-related macular degeneration. N Engl J Med.

[16] Rosenfeld PJ (2006). Ranibizumab for neovascular age-related macular degeneration. N Engl J Med.

[17] Solomon SD (2019). Anti-vascular endothelial growth factor for neovascular age-related macular degeneration. Cochrane Database Syst Rev.

[18] Folk JC, Stone EM (2010). Ranibizumab therapy for neovascular age-related macular degeneration. N Engl J Med.

[19] Krüger Falk M, Kemp H, Sørensen TL (2013). Four-year treatment results of neovascular age-related macular degeneration with ranibizumab and causes for discontinuation of treatment. Am J Ophthalmol.

[20] Rofagha S (2013). Seven-year outcomes in ranibizumab-treated patients in ANCHOR, MARINA, and HORIZON: a multicenter cohort study (SEVEN-UP). Ophthalmology.

[21] Chakravarthy U (2017). Phase I trial of anti-vascular endothelial growth Factor/Anti-angiopoietin 2 Bispecific Antibody RG7716 for Neovascular Age-Related Macular Degeneration. Ophthalmol Retina.

[22] Liberski S, Wichrowska M, Kocięcki J. Aflibercept versus Faricimab in the treatment of neovascular age-related macular degeneration and diabetic macular edema: a review. Int J Mol Sci. 2022;23(16). 10.3390/ijms23169424 PMC9409486 36012690

[23] Barnett JM, Hubbard GB (2021). Complications of retinopathy of prematurity treatment. Curr Opin Ophthalmol.

[24] Gossop M, Strang J, Bradley B (1989). The Bethlem study on NET: putting the record straight. Br J Addict.

[25] Giannaccare G (2020). Anti-VEGF treatment in corneal diseases. Curr Drug Targets.

[26] Fabre M et al. Recent advances in age-related macular degeneration therapies. Molecules. 2022;27(16). 10.3390/molecules27165089 PMC9414333 36014339

[27] Anderson DM (2015). A micropeptide encoded by a putative long noncoding RNA regulates muscle performance. Cell.

[28] Ruan X (2020). In vivo functional analysis of non-conserved human lncRNAs associated with cardiometabolic traits. Nat Commun.

[29] Lasda E, Parker R (2014). Circular RNAs: diversity of form and function. RNA.

[30] Quinn JJ, Chang HY (2016). Unique features of long non-coding RNA biogenesis and function. Nat Rev Genet.

[31] Schmitz SU, Grote P, Herrmann BG (2016). Mechanisms of long noncoding RNA function in development and disease. Cell Mol Life Sci.

[32] Kopp F, Mendell JT (2018). Functional classification and experimental dissection of long noncoding RNAs. Cell.

[33] Yu B, Wang S (2018). Angio-LncRs: LncRNAs that regulate angiogenesis and vascular disease. Theranostics.

[34] Wang KC (2011). A long noncoding RNA maintains active chromatin to coordinate homeotic gene expression. Nature.

[35] Kim K (2008). Isolation and characterization of a novel H1.2 complex that acts as a repressor of p53-mediated transcription. J Biol Chem.

[36] Jaé N, Dimmeler S (2020). Noncoding RNAs in Vascular diseases. Circ Res.

[37] Sauvageau M (2013). Multiple knockout mouse models reveal lincRNAs are required for life and brain development. Elife.

[38] Han X (2018). Mouse knockout models reveal largely dispensable but context-dependent functions of lncRNAs during development. J Mol Cell Biol.

[39] Liu JY (2014). Pathogenic role of lncRNA-MALAT1 in endothelial cell dysfunction in diabetes mellitus. Cell Death Dis.

[40] Cremer S (2019). Hematopoietic Deficiency of the long noncoding RNA MALAT1 promotes atherosclerosis and plaque inflammation. Circulation.

[41] Gast M (2019). Immune system-mediated atherosclerosis caused by deficiency of long non-coding RNA MALAT1 in ApoE-/-mice. Cardiovasc Res.

[42] Yang L (2021). Ablation of lncRNA. Theranostics.

[43] Tang X et al. Long noncoding RNA LEENE promotes angiogenesis and ischemic recovery in diabetes models. J Clin Invest. 2023;133(3). 10.1172/JCI161759 PMC9888385 36512424

[44] Zhang L (2020). Altered long non-coding RNAs involved in Immunological Regulation and Associated with Choroidal Neovascularization in mice. Int J Med Sci.

[45] Zhang P (2020). LncRNA NEAT1 sponges MiRNA-148a-3p to suppress Choroidal neovascularization and M2 macrophage polarization. Mol Immunol.

[46] Zhang L (2019). Microarray analysis of long non-coding RNAs and Messenger RNAs in a mouse model of Oxygen-Induced Retinopathy. Int J Med Sci.

[47] Wang Y (2020). Expression profiles of long noncoding RNAs in retinopathy of prematurity. Neural Regen Res.

[48] Yan B (2014). Aberrant expression of long noncoding RNAs in early diabetic retinopathy. Invest Ophthalmol Vis Sci.

[49] Zhao C (2019). Long non-coding RNA HEIH contributes to diabetic retinopathy by regulating miR-939/VEGF axis. Int J Clin Exp Pathol.

[50] Shan K (2016). Role of long non-coding RNA-RNCR3 in atherosclerosis-related vascular dysfunction. Cell Death Dis.

[51] Shan K (2017). RNCR3: a regulator of diabetes mellitus-related retinal microvascular dysfunction. Biochem Biophys Res Commun.

[52] Shi Y (2019). LncRNA FENDRR promotes high-glucose-induced proliferation and angiogenesis of human retinal endothelial cells. Biosci Biotechnol Biochem.

[53] Biswas S (2022). Expressions of serum lncRNAs in Diabetic Retinopathy - a potential Diagnostic Tool. Front Endocrinol (Lausanne).

[54] Dai R (2022). LncRNA LUADT1 inhibits cell apoptosis in diabetic retinopathy by regulating miR-383/peroxiredoxin 3 axis. Arch Physiol Biochem.

[55] Cataldi S et al. Diabetic retinopathy: are lncRNAs new molecular players and targets? Antioxidants (Basel). 2022;11(10). 10.3390/antiox11102021 PMC9598326 36290744

[56] Simion V, Haemmig S, Feinberg MW (2019). LncRNAs in vascular biology and disease. Vascul Pharmacol.

[57] Thomas AA, Feng B, Chakrabarti S (2017). ANRIL: A Regulator of VEGF in Diabetic Retinopathy. Invest Ophthalmol Vis Sci.

[58] Wei JC, Shi YL, Wang Q (2019). LncRNA ANRIL knockdown ameliorates retinopathy in diabetic rats by inhibiting the NF-kappaB pathway. Eur Rev Med Pharmacol Sci.

[59] Guan J (2022). Babao Dan inhibits lymphangiogenesis of gastric cancer in vitro and in vivo via lncRNA-ANRIL/VEGF-C/VEGFR-3 signaling axis. Biomed Pharmacother.

[60] Zhang B (2017). Overexpression of lncRNA ANRIL up-regulates VEGF expression and promotes angiogenesis of diabetes mellitus combined with cerebral infarction by activating NF-kappaB signaling pathway in a rat model. Oncotarget.

[61] Ruiz MA, Feng B, Chakrabarti S (2015). Polycomb repressive complex 2 regulates MiR-200b in retinal endothelial cells: potential relevance in diabetic retinopathy. PLoS ONE.

[62] Choi YC (2011). Regulation of vascular endothelial growth factor signaling by miR-200b. Mol Cells.

[63] Huang Q (2020). Overexpression of long non-coding RNA ANRIL promotes post-ischaemic angiogenesis and improves cardiac functions by targeting Akt. J Cell Mol Med.

[64] Chuang CC et al. Association of long noncoding RNA HOTAIR polymorphism and the clinical manifestations of diabetic retinopathy. Int J Environ Res Public Health. 2022;19(21). 10.3390/ijerph192114592 PMC9658836 36361470

[65] Rajagopal T (2020). HOTAIR LncRNA: a novel oncogenic propellant in human cancer. Clin Chim Acta.

[66] Zhao D (2020). Long noncoding RNA hotair facilitates retinal endothelial cell dysfunction in diabetic retinopathy. Clin Sci (Lond).

[67] Biswas S (2021). The long non-coding RNA HOTAIR is a critical epigenetic mediator of Angiogenesis in Diabetic Retinopathy. Invest Ophthalmol Vis Sci.

[68] Tsai MC (2010). Long noncoding RNA as modular scaffold of histone modification complexes. Science.

[69] Lian Y (2016). HOTTIP: a critical oncogenic long non-coding RNA in human cancers. Mol Biosyst.

[70] Sun Y, Liu YX (2018). LncRNA HOTTIP improves diabetic retinopathy by regulating the p38-MAPK pathway. Eur Rev Med Pharmacol Sci.

[71] Yang J (2021). LncRNA H19: a novel oncogene in multiple cancers. Int J Biol Sci.

[72] Venkatraman A (2013). Maternal imprinting at the H19-Igf2 locus maintains adult haematopoietic stem cell quiescence. Nature.

[73] Jia P (2016). Long non-coding RNA H19 regulates glioma angiogenesis and the biological behavior of glioma-associated endothelial cells by inhibiting microRNA-29a. Cancer Lett.

[74] Liu ZZ (2020). LncRNA H19 promotes glioma angiogenesis through miR-138/HIF-1α/VEGF axis. Neoplasma.

[75] Sun B et al. Long non-coding RNA H19 promotes corneal neovascularization by targeting microRNA-29c. Biosci Rep. 2019;39(5). 10.1042/BSR20182394 PMC6499455 30948500

[76] Thomas AA (2019). lncRNA H19 prevents endothelial-mesenchymal transition in diabetic retinopathy. Diabetologia.

[77] Luo R (2020). lncRNA H19 sponging miR-93 to regulate inflammation in retinal epithelial cells under hyperglycemia via XBP1s. Inflamm Res.

[78] Luo R (2021). LncRNA H19 inhibits high glucose-induced inflammatory responses of human retinal epithelial cells by targeting miR-19b to increase SIRT1 expression. Kaohsiung J Med Sci.

[79] Fawzy MS (2020). Circulating long noncoding RNAs H19 and GAS5 are associated with type 2 diabetes but not with diabetic retinopathy: a preliminary study. Bosn J Basic Med Sci.

[80] Wevrick R, Kerns JA, Francke U (1994). Identification of a novel paternally expressed gene in the Prader-Willi syndrome region. Hum Mol Genet.

[81] Stelzer Y (2014). The noncoding RNA IPW regulates the imprinted DLK1-DIO3 locus in an induced pluripotent stem cell model of Prader-Willi syndrome. Nat Genet.

[82] Yang TJ (2021). Suppression of choroidal neovascularization by silencing of long non-coding RNA IPW. Aging.

[83] Gu Y (2019). miR-370 inhibits the angiogenic activity of endothelial cells by targeting smoothened (SMO) and bone morphogenetic protein (BMP)-2. FASEB J.

[84] Ji P (2003). MALAT-1, a novel noncoding RNA, and thymosin beta4 predict metastasis and survival in early-stage non-small cell lung cancer. Oncogene.

[85] Chen Z (2022). LncRNA MALAT1 aggravates the retinal angiogenesis via miR-320a/HIF-1alpha axis in diabetic retinopathy. Exp Eye Res.

[86] Tan AJ et al. Knockdown of Malat1 alleviates high-glucose-induced angiogenesis through regulating miR-205-5p/VEGF-A axis. Exp Eye Res. 2021;207. 10.1016/j.exer.2021.108585 33887222

[87] Xia FJ et al. Competing endogenous RNA network associated with oxygen-induced retinopathy: expression of the network and identification of the MALAT1/miR-124-3p/EGR1 regulatory axis. Exp Cell Res. 2021;408(1). 10.1016/j.yexcr.2021.112783 34469714

[88] Yu L (2020). Long noncoding RNA MALAT1 participates in the pathological angiogenesis of diabetic retinopathy in an oxygen-induced retinopathy mouse model by sponging miR-203a-3. Can J Physiol Pharmacol.

[89] Michalik KM (2014). Long noncoding RNA MALAT1 regulates endothelial cell function and vessel growth. Circ Res.

[90] Zhang YL (2020). Targeting long non-coding RNA MALAT1 alleviates retinal neurodegeneration in diabetic mice. Int J Ophthalmol.

[91] Wang Y et al. Effect and mechanism of the long noncoding RNA MALAT1 on retinal neovascularization in retinopathy of prematurity. Life Sci. 2020;260. 10.1016/j.lfs.2020.118299 32827542

[92] Ghafouri-Fard S, Azimi T, Taheri M (2021). Myocardial infarction Associated transcript (MIAT): review of its impact in the tumorigenesis. Biomed Pharmacother.

[93] Li Q (2018). Long non-coding RNA of myocardial infarction Associated transcript (LncRNA-MIAT) promotes Diabetic Retinopathy by upregulating transforming growth Factor-β1 (TGF-β1) signaling. Med Sci Monit.

[94] Yan B (2015). lncRNA-MIAT regulates microvascular dysfunction by functioning as a competing endogenous RNA. Circ Res.

[95] Zhang J et al. Long non-coding RNA MIAT acts as a biomarker in diabetic retinopathy by absorbing. Biosci Rep. 2017;37(2). 10.1042/BSR20170036 PMC5408653 28246353

[96] Yu C (2020). Downregulation of long noncoding RNA MIAT in the retina of Diabetic rats with tail-vein injection of human umbilical-cord mesenchymal stem cells. Int J Med Sci.

[97] Liu CH (2015). Endothelial microRNA-150 is an intrinsic suppressor of pathologic ocular neovascularization. Proc Natl Acad Sci U S A.

[98] An H. NEAT1 and paraspeckles in neurodegenerative diseases: a missing lnc found? (vol 3, pg 243, 2018). Non-Coding Rna Research. 2020; 5(4):219–219. 10.1016/j.ncrna.2018.11.003 PMC6257911 30533572

[99] Ge ZW et al. Long noncoding RNA NEAT1 promotes cardiac fibrosis in heart failure through increased recruitment of EZH2 to the Smad7 promoter region. J Translational Med. 2022;20(1). 10.1186/s12967-021-03211-8 PMC8722118 34980170

[100] Li XJ (2018). Long non-coding RNA nuclear paraspeckle assembly transcript 1 inhibits the apoptosis of retina Müller cells after diabetic retinopathy through regulating miR-497/brain-derived neurotrophic factor axis. Diab Vasc Dis Res.

[101] Shao K (2020). Knockdown of NEAT1 exerts suppressive effects on diabetic retinopathy progression via inactivating TGF-β1 and VEGF signaling pathways. J Cell Physiol.

[102] Yang Y (2021). Regulated diabetic retinal epithelial-mesenchymal transition through regulating miR-204/SOX4 axis. PeerJ.

[103] Xu Y (2021). LncRNA NEAT1 promotes gastric Cancer Progression through miR-17-5p/TGFbetaR2 Axis Up-Regulated angiogenesis. Front Cell Dev Biol.

[104] Li J (2016). LncRNA TUG1 acts as a tumor suppressor in human glioma by promoting cell apoptosis. Exp Biol Med (Maywood).

[105] Li Z (2016). TUG1: a pivotal oncogenic long non-coding RNA of human cancers. Cell Prolif.

[106] Cai H (2017). Long non-coding RNA taurine upregulated 1 enhances tumor-induced angiogenesis through inhibiting microRNA-299 in human glioblastoma. Oncogene.

[107] Dong R (2016). Targeting long non-coding RNA-TUG1 inhibits tumor growth and angiogenesis in hepatoblastoma. Cell Death Dis.

[108] Liu L et al. Long non-coding RNA TUG1 promotes endometrial cancer development via inhibiting miR-299 and miR-34a-5p | Liu Oncotarget. 2017. 10.18632/oncotarget.15607 PMC5458215 28404901

[109] Yuan MX et al. lncRNA TUG1 regulates angiogenesis via the miR–204–5p/JAK2/STAT3 axis in hepatoblastoma. Mol Med Rep. 2021;24(2). 10.3892/mmr.2021.12192 34080023

[110] Wang Y (2022). LncRNA TUG1 promotes apoptosis, Invasion, and angiogenesis of retinal endothelial cells in retinopathy of Prematurity via MiR-145-5. Front Med (Lausanne).

[111] Li Y et al. TUG1 enhances high glucose-impaired endothelial progenitor cell function via miR-29c-3p/PDGF-BB/Wnt signaling. Stem Cell Res Ther. 2020;11(1):441. 10.1186/s13287-020-01958-3 PMC7558752 33059750

[112] Zhou Y (2007). Activation of p53 by MEG3 non-coding RNA. J Biol Chem.

[113] Zhou Y, Zhang X, Klibanski A (2012). MEG3 noncoding RNA: a tumor suppressor. J Mol Endocrinol.

[114] Qiu GZ (2016). Long noncoding RNA-MEG3 is involved in diabetes mellitus-related microvascular dysfunction. Biochem Biophys Res Commun.

[115] Ruan W (2018). Knockdown of long noncoding RNA MEG3 impairs VEGF-stimulated endothelial sprouting angiogenesis via modulating VEGFR2 expression in human umbilical vein endothelial cells. Gene.

[116] Zhang D (2018). LncRNA MEG3 overexpression inhibits the development of diabetic retinopathy by regulating TGF-β1 and VEGF. Exp Ther Med.

[117] Chen J (2021). Long non-coding RNA MEG3 inhibits neovascularization in diabetic retinopathy by regulating microRNA mir-6720-5p and cytochrome B5 reductase 2. Bioengineered.

[118] He C (2017). Long noncoding RNA MEG3 negatively regulates proliferation and angiogenesis in vascular endothelial cells. DNA Cell Biol.

[119] Andersen RE (2019). The long noncoding RNA pnky is a trans-acting Regulator of cortical development in vivo. Dev Cell.

[120] Shi L (2022). Long non-coding RNA PNKY modulates the development of Choroidal Neovascularization. Front Cell Dev Biol.

[121] Ramos AD (2015). The long noncoding RNA pnky regulates neuronal differentiation of embryonic and postnatal neural stem cells. Cell Stem Cell.

[122] Li X (2021). Long non-coding RNA SNHG16 regulates E2F1 expression by sponging miR-20a-5p and aggravating proliferative diabetic retinopathy. Can J Physiol Pharmacol.

[123] Cai F (2021). Upregulation of long non-coding RNA SNHG16 promotes diabetes-related RMEC dysfunction via activating NF-kappaB and PI3K/AKT pathways. Mol Ther Nucleic Acids.

[124] Zhang R (2021). Decreased lncRNA SNHG16 accelerates oxidative stress Induced Pathological Angiogenesis in Human Retinal Microvascular endothelial cells by regulating miR-195/mfn2 Axis. Curr Pharm Des.

[125] Wang HS (2020). Long noncoding RNA SNHG6 promotes proliferation and angiogenesis of cholangiocarcinoma cells through sponging mir-101-3p and activation of E2F8. J Cancer.

[126] Zhao W (2018). LncRNA SNHG16 drives proliferation, migration, and invasion of hemangioma endothelial cell through modulation of miR-520d-3p/STAT3 axis. Cancer Med.

[127] Atef MM (2022). The evolving role of long noncoding RNA HIF1A-AS2 in diabetic retinopathy: a cross-link axis between hypoxia, oxidative stress and angiogenesis via MAPK/VEGF-dependent pathway. Redox Rep.

[128] Pan J, Zhao L (2021). Long non-coding RNA histone deacetylase 4 antisense RNA 1 (HDAC4-AS1) inhibits HDAC4 expression in human ARPE-19 cells with hypoxic stress. Bioengineered.

[129] Geng H (2011). HDAC4 protein regulates HIF1α protein lysine acetylation and cancer cell response to hypoxia. J Biol Chem.

[130] Seo HW (2009). Transcriptional activation of hypoxia-inducible factor-1alpha by HDAC4 and HDAC5 involves differential recruitment of p300 and FIH-1. FEBS Lett.

[131] Xu XD (2014). Long non-coding RNAs: new players in ocular neovascularization. Mol Biol Rep.

[132] Li F (2016). Novel insights into the role of long noncoding RNA in Ocular diseases. Int J Mol Sci.

[133] Awan HM (2017). Primate-specific long non-coding RNAs and MicroRNAs. Genomics Proteom Bioinf.

[134] Zhou Q et al. LncEGFL7OS regulates human angiogenesis by interacting with MAX at the EGFL7/miR-126 locus. Elife. 2019;8. 10.7554/eLife.40470 PMC6370342 30741632

[135] Amati B, Land H (1994). Myc-Max-Mad: a transcription factor network controlling cell cycle progression, differentiation and death. Curr Opin Genet Dev.

[136] Vervoorts J (2003). Stimulation of c-MYC transcriptional activity and acetylation by recruitment of the cofactor CBP. EMBO Rep.

[137] Pichler M (2020). Therapeutic potential of FLANC, a novel primate-specific long non-coding RNA in colorectal cancer. Gut.

[138] Menaa F (2017). Sickle cell retinopathy: improving care with a multidisciplinary approach. J Multidiscip Healthc.

[139] Li Z (2022). HOTTIP mediated Therapy Resistance in Glioma cells involves regulation of EMT-Related miR-10b. Front Oncol.

[140] Wei H (2022). Long non-coding RNA PAARH promotes hepatocellular carcinoma progression and angiogenesis via upregulating HOTTIP and activating HIF-1α/VEGF signaling. Cell Death Dis.

[141] Yuan X, Sun Z, Cui C (2021). Knockdown of lncRNA HOTTIP inhibits Retinoblastoma Progression by modulating the miR-101-3p/STC1 Axis. Technol Cancer Res Treat.

